# CD109 acts as a gatekeeper of the epithelial trait by suppressing epithelial to mesenchymal transition in squamous cell carcinoma cells *in vitro*

**DOI:** 10.1038/s41598-019-50694-z

**Published:** 2019-11-06

**Authors:** Shufeng Zhou, Sabrina Daniela da Silva, Peter M. Siegel, Anie Philip

**Affiliations:** 1 Division of Plastic Surgery, Department of Surgery, McGill University, Montreal, Canada; 2 Department of Otolaryngology – Head and Neck Surgery, McGill University, Montreal, Canada; 3 Rosalind and Morris Goodman Cancer Research Centre, Faculty of Medicine, McGill University, Montreal, Canada

**Keywords:** Squamous cell carcinoma, Oral cancer detection

## Abstract

There is increasing evidence that the expression of CD109, a GPI-anchored cell surface protein is dysregulated in squamous cell carcinoma (SCC). However, the functional role of CD109 in SCC progression is poorly understood. In current study, we demonstrate that CD109 is a critical regulator of epithelial phenotype in SSC cells. CD109 levels inversely correlate with TGF-β signaling, EMT, migration, and invasion in cultured SCC cells. CRISPR/Cas9-mediated knockout CD109 (CD109 KO) in SCC cells represses epithelial traits and promotes the mesenchymal phenotype, as evidenced by elevated expression of mesenchymal proteins and markers of epithelial to mesenchymal transition. Treatment with recombinant CD109 protein causes CD109 KO cells to regain their epithelial traits. CD109 loss results in pronounced alterations of gene expression as detected by microarray analysis and in dysregulation of 15 important signalling pathways as shown by KEGG pathway cluster analysis. Validation using 52 human oral SCC tumor samples show that CD109 levels inversely correlate with tumor grade and the activation state of one such pathway, the TGF-β signaling pathway. Taken together, our findings highlight a novel role for CD109 as a gatekeeper of the epithelial phenotype by regulating TGF-β pathway in SCC cells.

## Introduction

Squamous cell carcinoma (SCC) is one of the leading causes of cancer-related death worldwide^1^. During the past 30 years, the incidence of SCC has been rising by 3 to 10% per year and represents one third of all new cancer cases each year in North America^2^. SCCs have a high tendency to metastasize and approximately 16% of SCCs progress to metastatic disease^3^. Although an epithelial-to-mesenchymal transition (EMT), by which epithelial cells lose epithelial traits and acquire a mesenchymal phenotype, is a normal developmental process, it is well known to play a critical role in the conversion of early-stage SCC tumours to invasive malignancies^4,5^.

Transforming growth factor-beta (TGF-β) is a potent EMT inducer and promotes tumor cell migration and invasion^6^. CD109 is a glycosylphosphatidylinositol (GPI)-anchored protein that our group has identified as a TGF-β co-receptor and a potent inhibitor of the TGF-β signalling^7–9^. The expression of CD109 is dysregulated in SCC^10–13^ and in several tumors^14–17^. Previous studies revealed a negative correlation between CD109 expression and advanced SCC tumor grade^13^. Notably, CD109 is highly expressed in well-to-moderate SCC relative to poorly differentiated SCC tumors, suggesting that CD109 might play an essential role in tumor progression. Recently, we showed that CD109 can be released from human bone marrow mesenchymal stem cells and attenuates TGF-β-induced EMT and stemness of SCC cells^18^. Despite the increasing evidence indicating a potential role for CD109 in SCC, the molecular mechanisms by which CD109 regulates SCC progression and metastasis remain poorly understood. In the present study, we show that CD109 is heterogeneously expressed in SCC cells, and cellular levels of CD109 are inversely correlated with EMT markers expression, migration, and invasion. Furthermore, we demonstrate that CRISPR/Cas9-mediated knockout of CD109 induces morphologic and molecular changes indicative of enhanced EMT traits. Further validations using 52 human oral SCC samples show that CD109 levels inversely correlate with TGF-β signalling and tumor grade. In summary, CD109 functions as a “gatekeeper” of the epithelial phenotype and represses mesenchymal traits through modulation of the canonical TGF-β pathway to inhibit EMT.

## Results

### CD109 levels are inversely correlated with TGF-β signaling, pathological grade and tumor stage in human oral squamous cell carcinomas

To explore the clinical relevance of CD109 in human oral SCC, we assessed the expression of CD109, TGF-β and phosphoSmad2 (P-Smad2) in 52 human oral SCC (OSCC) tumor samples using tissue microarray immunohistochemistry. We analyzed a cohort of 52 patients, 31 males (59.6%) and 21 females (40.4%), with a mean age of 55.2 years (range 40–89). Alcohol consumption was observed in 27 patients (72.8%) and tobacco smoking in 25 (52.1%). A total of 32 cases (61.5%) had early clinical stage (T1 + T2), while 20 (38.5%) presented at advanced clinical stage (T3 + T4). Twelve patients (23.1%) had a lymph node metastatic disease (pN+) (Supplemental Table 1).

**Figure 1:**
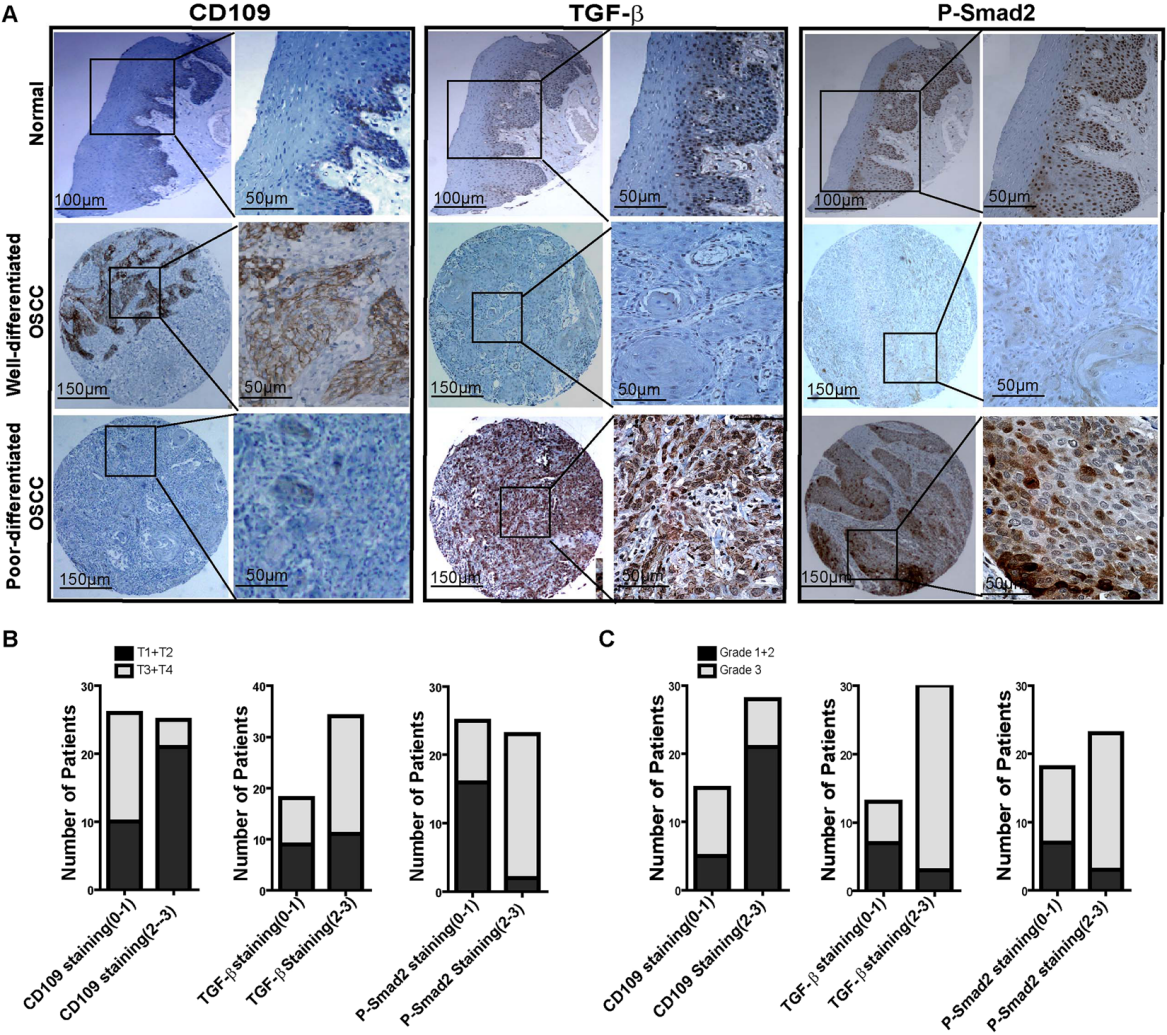
CD109 expression inversely correlates with TGF-β/Smad signalling, tumor clinical stages and tumor grades in OSCC. (**A**) Representative IHC image of CD109, TGF-β, and P-Smad2 expression in normal oral epithelium (Top), well-differentiated (Middle) or poorly-differentiated (Bottom) OSCC as indicated. An inverse relationship between CD109 expression and TGF-β/P-Smad2 in OSCC tissues was observed. (**B**) Distribution of the clinical stages relative to CD109, TGF-β and P-Smad2 protein expression in OSCC. Patient with high staining of CD109 (Score 2–3) were associated with early clinical stage (T1 + T2) whereas patient with high staining of TGF-β and P-Smad2 were associated with advanced clinical stage (T3 + T4). (**C**) Distribution of the tumor grade relative to CD109, TGF-β and P-Smad2 protein expression in OSCC. High staining of CD109 (Score 2–3) were associated with well-differentiated status (Grade I + II) whereas high level of TGF-β and P-Smad2 were associated with poorly-differentiated status (Grade III). Chi-square has been used to find the significance of study parameters on categorical scale between two or more groups. A value of P < 0.05 was considered statistically significant. All analyses were performed with Prism GraphPad.

As shown by IHC staining (Fig. 1A), CD109 expression was barely detectable in normal epithelia (Fig. 1A top) but was strongly expressed in the well-differentiated OSCC concomitantly with weak expression of TGF-β and P-Smad2 (Fig. 1A middle). In contrast, poorly differentiated tumors exhibited weakly staining for CD109, but markedly intense staining for both TGF-β and P-Smad2 (Fig. 1A bottom). These observations suggest an inverse relationship between CD109 expression and TGF-β/P-Smad2 levels in OSCC tissues.

To confirm this observation, we assessed the correlation between CD109, TGF-β and P-Smad2 in relation to tumor clinical stages and pathological grades. As summarized in Table 1, about 41% of early clinical stage (T1 + T2) patients’ tumors exhibited strong CD109 expression, whereas only 7.8% of late stage (T3 + T4) patients’ tumors had strong expression of CD109 (χ^2^ test: *P* = 0.01), indicating that CD109 expression was markedly higher in early clinical stage OSCC tumors. Consistent with this result, CD109 expression was significantly higher in low grade OSCC (Grade I + II) than in high grade tumors (Grade III). About 48% of lower grade tumors (Grade I + II) exhibited strong CD109 expression while only 16.3% of Grade III tumors stained strong for CD109 (χ^2^ test: *P* < 0.01). Altogether, the above results suggest that strong CD109 staining is associated with early clinical stage and low-grade tumor in OSCC patients.

**Table 1:** Correlation Among Protein Staining with Clinical Stage and Histologic Grade in OSCC patients.

Variables	Tumor Classification No. (%)				Histological Grade No. (%)			
	T1 + T2	T3 + T4	P	OR (95% CI)	Grade I + II	Grade III	P	OR (95% CI)
CD109								
Weakly (0–1)	10 (19.6%)	16 (31.4%)	0.01	0.12 (0.03–0.45)	5 (11.6%)	10 (23.3%)	0.01	0.1667 (0.042–0.658)
Strongly (2–3)	21 (41.2%)	4 (7.8%)			21 (48.8%)	7 (16.3%)		
TGF-β								
Weakly (0–1)	9 (17.3%)	9 (17.3%)	0.21	2.09 (0.649–6.74)	7 (16.2%)	3 (7.0%)	0.01	10.50 (2.085–52.87)
Strongly (2–3)	11 (21.2%)	23 (44.2%)			6 (14.0%)	27 (62.8%)		
P-Smad2								
Weakly (0–1)	16 (33.3%)	9 (18.8%)	0.001	18.67 (3.53–98.66)	7 (17.1%)	11 (26.8%)	0.05	4.242 (2.91–19.79)
Strongly (2–3)	2 (4.2%)	21 (43.7%)			3 (7.3%)	20 (48.8%)		

In contrast, as shown in the Table 1/Fig. 1, TGF-β staining is significantly higher in poorly differentiated tumors when compared to well-differentiated tumors. More than 62.8% of high-grade tumors (Grade III) expressed high levels of TGF-β while only 14% low grade (Grade I + II) tumors showed similar expression levels of TGF-β. (χ^2^ test: *P* = 0.01). When correlated with the clinical stage, we observed that strong TGF-β stains preferentially in advanced stages of OSCC. Thus, 44.2% of late clinical stage tumors (T3 + T4) expressed high levels of TGF-β compared to 21.2% of early clinical stage tumors (T1 + T2). Although we observed a clear association of strong TGF–β expression with advanced tumor, the size of our cohort was too small to reach statistical significance (χ^2^ test: *P* = 0.2). Similarly, the expression of P-Smad2 was found to be significantly higher in late clinical stage of tumors. About 43% of advanced clinical stage tumors (T3 + T4) exhibited strong P-Smad2 staining, whereas only 4.2% of early clinical stage tumors (T1 + T2) exhibited similar expression levels (χ^2^ test: *P* = 0.001). A similar association was observed between tumor pathological grade and P-Smad2 expression: more that 48% of high grade (Grade III) tumors expressed high levels of P-Smad2 while only 7.3% of low grade (Grade I + II) tumors showed high levels of P-Smad2 expression (χ^2^ test: P = 0.05) (Table 1/Fig. 1B). These results suggest that TGF-β/P-Smad2 pathway activation is associated with high tumor grade and advanced clinical stage in OSCC patients.

Finally, we also calculated odds ratios of the association between CD109, TGF-β/P-Smad2, clinical stage and tumor grade in OSCC (Table 1). Patients with tumors staining strongly for CD109 were less likely to develop to advanced clinical stage (OR = 0.119; 95% CI = 0.031 to 0.45). Similarly, these patients were also less likely to develop poorly differentiated tumors (OR = 0.1667; 95% CI = 0.0422 to 0.6579). Conversely, patients with tumors strongly expressing TGF-β or P-Smad2 are more likely to be classified as advanced tumor stage (TGF-β: OR = 2.09; 95% CI = 0.6485 to 6.742) (P-Smad2: OR = 18.67; 95% CI 3.532 to 98.66). Similarly, these patients were more likely to develop poorly differentiated tumors (TGF-β: OR = 10.5; 95% CI 2.085 to 52.87; P-Smad2: OR = 4.24; 95% CI 2.9095 to 19.79). These observations are consistent with previous findings of others and us^10–16,19^ and support the notion that CD109 negatively regulates TGF-β/Smad signaling, as our group previously demonstrated^7–9^.

### CD109 is heterogeneously expressed in cultured SCC cells and its levels inversely correlate with TGF-β signaling, EMT marker expression, cellular migration, and invasion *in vitro*

**Figure 2:**
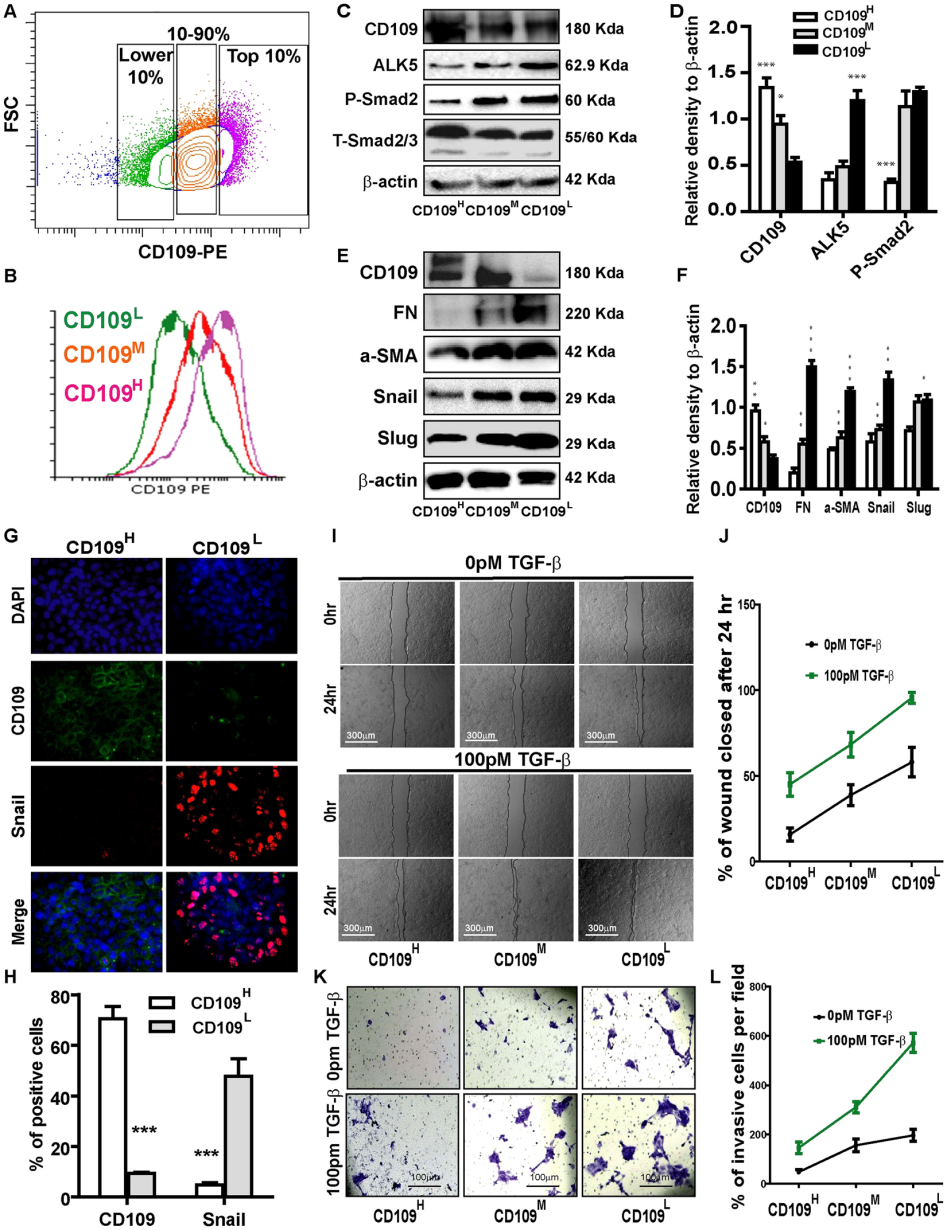
CD109 expression levels inversely correlate with TGF-β signaling, EMT marker expression, cellular migration and invasion. (**A**) Isolation of CD109^H^, CD109^M^, and CD109^L^ subpopulations of A431 SCC cells by flow cytometry based on their CD109 expression levels. (**B**) Sorted cells were put in culture for three weeks and then re-analyzed by flow cytometry for CD109 expression, which showing that they maintain their respective CD109 expression profiles. (**C**) Representative image and (**D**) quantification of Western blot analysis of TGF-β receptor I (ALK5) and P-Smad2 in CD109^H^, CD109^M^ CD109^L^ cells, showing that CD109 expression levels are inversely correlated with TGF-β signalling. (**E**) Representative image and (**F**) quantification of Western blot analysis for EMT markers in CD109^H^, CD109^M^, CD109^L^ cells, respectively. EMT markers expressions are inversely correlated with CD109 expression. (**G**) Representative image and (**H**) quantification of Immunofluorescence microscopy for CD109 (Green), Snail (Red) and DAPI (Blue) in CD109^H^ and CD109^L^ SCC cells, respectively, showing that CD109^H^ cells exhibited decreased Snail expression. (**I**) Representative images and (**J**) quantification of wound-healing assays on CD109^H^, CD109^M^, and CD109^L^ subpopulations as indicated, revealing that the levels of CD109 were inversely corelated with the migration of SCC cells. Cell migration was expressed as a percentage of the scratch area filled by migrating cells at 24 h post scratch: migration rate = (T0 hr scratch width − T24 hr scratch width)/T0 hr scratch width) × 100%. (**K**) Representative images and (**L**) quantification of an invasive assay done on equal number of CD109^H^, CD109^M^, and CD109^L^ subpopulations. 10,000 cells were seeded on a BioCoat™ Matrigel^®^ Invasion Chamber for 24 hours. Cells that invaded through the matrigel-coated membrane were stained with 1% crystal violet, photographed, and counted. The levels of CD109 are inversely corelated with cell invasion. All the results are expressed as the mean ± S.D. of three independent experiments. Significance is calculated using a One-Way ANOVA *P < 0.05. **P < 0.01 and ***P < 0.001. The graphs display the raw data. Scale bars: 30 μm, 100 μm and 300 μm, as indicated.

**Figure 3:**
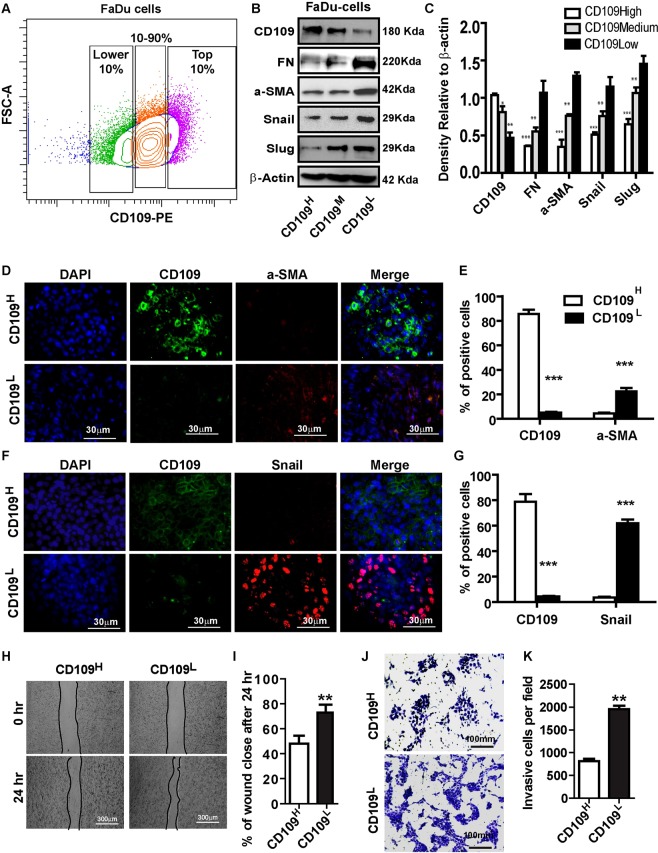
CD109 levels are inversely correlated with EMT marker expression, migration, invasion in FaDu cells. (**A**) Isolation of CD109^H^, CD109^M^, and CD109^L^ subpopulations of FaDu SCC cells by flow cytometry based on their CD109 expression levels. (**B**) Representative image and (**C**) quantification of Western blot for EMT markers in indicated samples, showing EMT markers expressions are inversely correlated with CD109 expression. (**D**,**F**) Representative images and (**E**,**G**) qualification of immunofluorescence microscopy stained for CD109 (green), a-SMA (red, **D**) and Snail (Red, **F**) and DAPI (blue) CD109^H^, or CD109^L^ FaDu cells, as indicated. a-SMA and Snail expressions were decreased in CD109^H^ FaDu cells. (**H**) Representative images and (**I**) quantification of wound healing assays. CD109 levels were inversely corelates with the motility of the FaDu cells. (**J**) Representative images and (**K**) quantification of an invasion assay. CD109 levels inversely corelated with the invasiveness of FaDu cells. All the results are expressed as the mean ± S.D. of three independent experiments. Significance is calculated using a one-way ANOVA; *P < 0.05. **P < 0.01 and ***P < 0.001. Magnification, ×100. Scale bars, 100 μm.

We next examined whether CD109 is heterogeneously expressed in 2 different SCC cell lines (A431 and FaDu). At 80% confluence, A431cells were sorted for CD109 expression into: CD109^high^ (Top 10%), CD109^low^ (Lower 10%) and CD109^Med^ (10–90%) (Fig. 2A). These sorted cells were maintained in culture for 3 weeks, then sorted again as above (Fig. 2B). A431 cells sorted in the three CD109 expression groups faithfully maintained this heterogeneity during subsequent culture (Fig. 2B). Interestingly, the levels of Alk5 (TGF-β receptor I), and P-Smad2 (downstream effector of TGF-β signalling) were markedly lower in CD109^high^ A431 cells compared to those in CD109^low^cells (Fig. 2C,D). To evaluate whether cellular levels of CD109 modulate EMT responses in SCCs, we measured expression of EMT markers in A431 cells and found that the expression of the mesenchymal proteins Fibronectin, α-SMA and the EMT transcription factors Snail, Slug, and Zeb2 were significantly attenuated in CD109^high^ A431cells (Fig. 2E,F). This was further confirmed by immunofluorescence microscopy showing that CD109^high^ cells exhibit markedly reduced mesenchymal proteins and EMT transcription factors expressions (Fig. 2G,H). We then evaluated the effect of CD109 levels on SCC cell migration by an in vitro wound healing assay and found that cell migration rates were significantly reduced in CD109^high^ *versus* CD109^low^ A431 cells, regardless of the presence or absence of exogeneous TGF-β (Fig. 2I,J). To determine the effect of CD109 on invasiveness, we carried out matrigel invasion assays. Our results demonstrate that CD109^high^ A431 cells exhibit a 2-fold reduction in cell invasion compared to CD109^low^ counterparts (Fig. 2K,L). To rule out the possibility that these results were specific to A431 cells, we repeated these experiments on FADU cells, a model cell line of oral squamous carcinoma and obtained comparable results as with the A431 cells (Fig. 3). Taken together, these observations demonstrate that SCC cells heterogeneously express CD109 and that CD109^low^ SCC cells exhibit enhanced TGF-β signaling, EMT marker expression as well as elevated cellular migration and invasion compared to CD109^high^ cells.

### Generation and verification of CD109-knockout A431 cell lines

**Figure 4:**
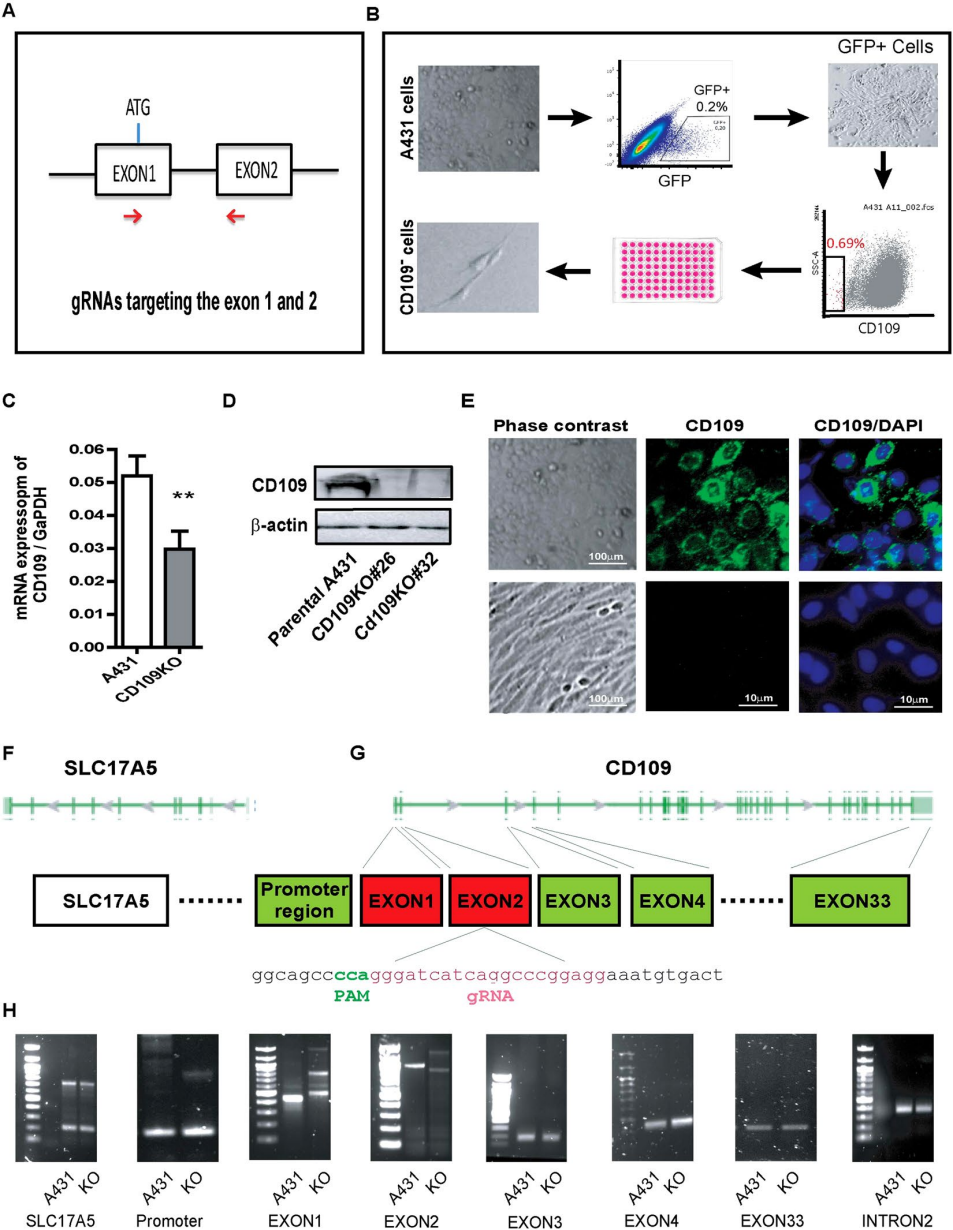
Generation and verification of the *CD109* knockout A431 cell lines by CRISPR/Cas9 editing. (**A**) Schematic map of the two sgRNAs that were designed to excise the Exon1 and Exon2 of the human CD109 gene. (**B**) Stepwise strategy to generate CD109 KO cell lines. (**C**) qPCR analysis of CD109 mRNA level in KO cells relative to parental A431 cells, showing CD109 mRNA level markedly decrease in CD109-KO cells. (**D**) Representative Western blot image of CD109-KO and parental A431 cells, showing non-detectable levels of CD109 protein in CD109 KO cells. (**E**) Phase contrast image of parental A431 (Top left) and CD109 KO cells (bottom left), demonstrating that CD109-KO cells acquired a mesenchymal phenotype and lose their epithelial trait. Immunofluorescence detection of CD109 in parental A431 (Top right) and CD109 KO (Bottom right), showed non-detectable CD109 staining in KO cells. (**F**,**G**) Schematic of CD109 genome and up-stream gene. (**H**) Characterization of genomic deletion at the CD109 locus by PCR.

To further investigate the role of CD109 in SCC cells, we used the CRISPR-Case9 gene editing system to generate stable *CD109*-knockout SCC cell lines^20^. gRNAs that target exon1 or exon2 of the *CD109* gene were designed (Fig. 4A) and CD109 negative cells were subsequently generated by single cell sorting to produce clonal CD109 KO cell lines (Fig. 4B). Although we worked on both A431and FaDu cells, we were able to successfully generate only A431 CD109 KO cell lines (CD109-KO) because FaDu cells were unable to survive the single cell sorting process. The efficacy of CD109 knockout was confirmed at the mRNA level (Fig. 4C) and protein level (Fig. 4D,E). We then characterized the mutations by PCR on the genomic DNA isolated from the selected single clones. The sizes of PCR products were different between parental and KO cells (Fig. 4F–H) which suggested that both exon 1 and exon 2 were mutated due to cas9-mediated DNA breaks at the *CD109* locus. We then performed PCR to detect exon 3, 4 and 33, intron 2, as well as the promoter region of CD109, and confirmed that these regions were not affected (Fig. 4G,H), suggesting the specificity of exon 1 or exon2 disruption. We also designed primers to detect *SLC17A5* the closest gene upstream to CD109 and found that it is present in the CD109 KO cells (Fig. 4F), suggesting that only the region encompassing exons 1 and 2 of *CD109* was deleted without affecting neighboring genes. Sequencing the PCR amplicons corresponding to exons 4 and 33 confirmed that these two exons were parts of the CD109 gene (Supplementary Fig. S1). Altogether, these results demonstrate that CD109-KO SCC A431cell lines were successfully generated by CRISPR/Cas9 and the mutation is in a frame deletion of exon1 and exon2 resulting in a truncated mutant without affecting the neighboring genomic regions.

### Loss of CD109 enhances cell proliferation and supresses apoptosis in SCC cells

**Figure 5:**
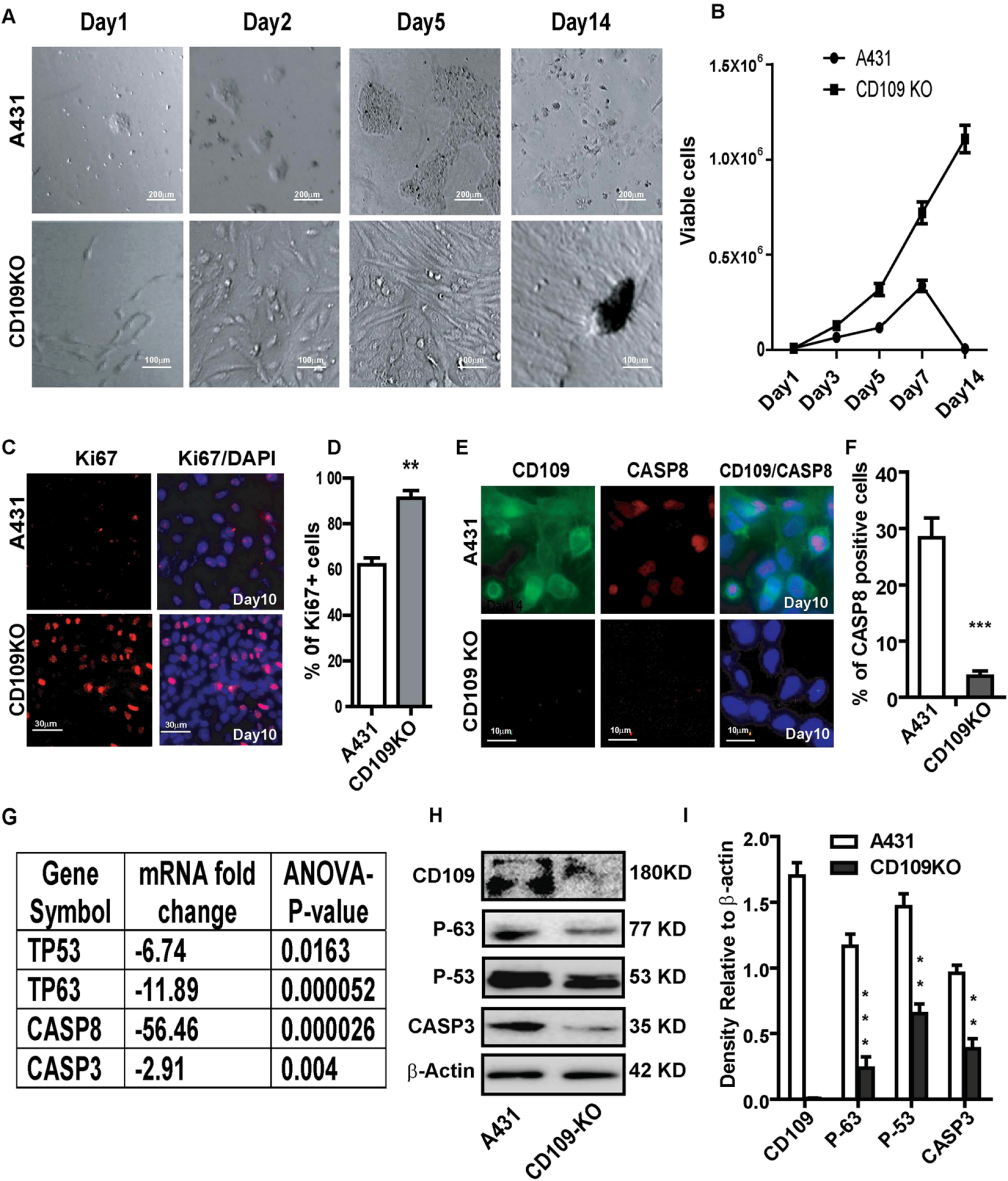
CD109-KO cells exhibited enhanced cell proliferation and suppressed apoptosis. (**A**) Representative Phase contrast pictures and (**B**) cell count analysis of parental and CD109-KO A431 cells at indicated time, showing that CD109 loss increases proliferation and decreases apoptosis. (**C**) Representative image and (**D**) qualification of Immunofluorescence microscopy for CD109 KO and A431 cells that stained for cell proliferate marker Ki67 (red) and DAPI (blue), revealing that CD109 loss significantly increased Ki67-positive cells. (**E**) Immunofluorescence microscopy and (**F**) qualification of CD109 KO and A431 cells stained for CD109 (green), apoptosis marker Caspase 8 (red), and DAPI (blue), indicating that CD109 loss significantly decreased Caspase 8 positive cells. (**G**) Microarray analysis shows that TP53, TP63, CASP3 and CASP8 are significantly down-regulated in CD109-KO cells. (**H**) Representative Western blot image and (**I**) quantification for CD109, TP53, TP63 and CASP3 in A431 and CD109-KO cells, confirming a decrease of these proteins in CD109-KO cells. β-actin was used as a protein loading control. All results are shown as the mean ± SD of at least three independent experiments. Significance is calculated using a one-way ANOVA analysis. *P < 0.05, **P < 0.01 and ***P < 0.001. Scale bars: 10 and 30 μm in low and high magnification, as indicated.

Interestingly, we observed that CD109-KO cells consistently demonstrate dramatically enhanced proliferation and supressed apoptosis even at high cell density, as evidenced by the cumulative growth curve (Fig. 5A,B). In contrast, parental A431 cells dramatically underwent apoptosis at full confluence, resulting in massive cell death, as indicated by decreased number of viable cells (Fig. 5A,B) as well as decreased ki67 (Fig. 5C,D) and increased Caspase 8 expressions when compared to CD109 KO cells (Fig. 5E,F). Importantly, CD109-KO cells showed no evidence of cell death/apoptosis and continually proliferated without cell death and apoptosis, resulting in multiple-layered cell growth (Fig. 5A). Furthermore, both mRNA and protein levels of tumour suppressors TP53 and TP63, which are known to promote apoptosis, and Caspase 3, were significantly downregulated in CD109 KO cells (Fig. 5G–I). These results suggest that loss of CD109 enhances proliferation and suppresses apoptosis by decreasing the expression of TP53, TP63 and Caspase family genes in SCC *in vitro*.

### CD109 KO SCC cells acquire a mesenchymal phenotype, and exhibit enhanced TGF-β signaling, EMT, motility and invasiveness

**Figure 6:**
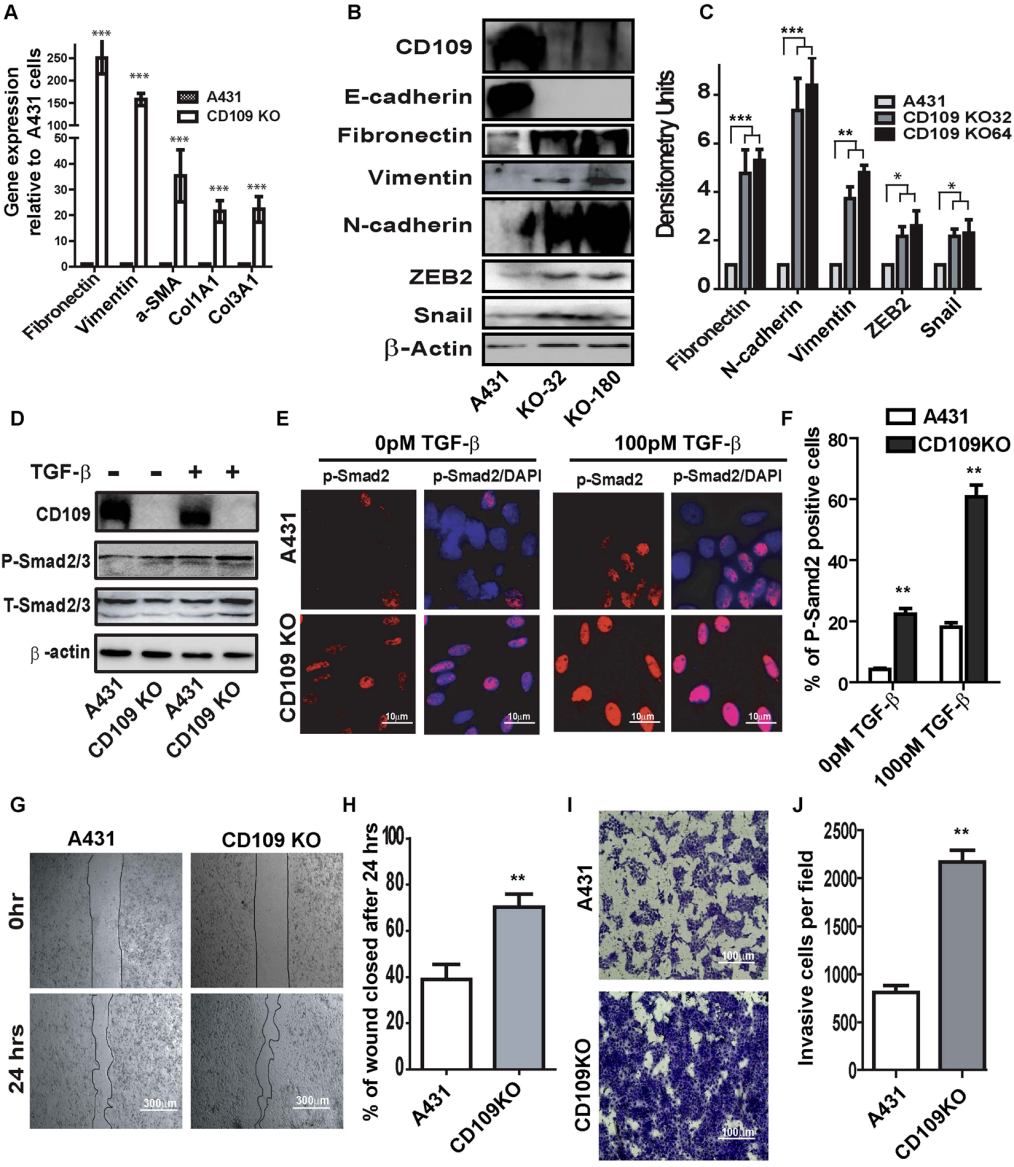
CD109 loss promotes EMT process, motility and invasion of SCCs. (**A**) Expression analysis by qPCR of fibronectin (FN), vimentin (VIM), a-SMA, Collagen 1A1 (Col1A1) and Collagen 3A1 (Col3A1). Results were first normalized with the housekeeping gene GAPDH, and the expressions in parental A431 cells were set at 1. Results are presented as mean ± SD (n = 3 independent experiments repeated in triplicates). (**B**) Representative Western blot image and (**C**) quantification of EMT markers. CD109 loss significantly enhanced the EMT marker expressions. (**D**) Representative images of Western blot and (**E**) Immunofluorescence microscopy and (**F**) quantification for P-Smad2 expression in A431 and CD109 KO A431 cells, showing an enhanced p-Smad2 activity in the CD109-KO cells. (**G**) Representative images and (**H**) quantification of wound-healing assays on parental and CD109 KO A431 cells, indicating CD109 loss enhanced the migration of SCC cells. Cell migration is expressed as a percentage of the scratch area filled by migrating cells at 24 h post scratch: migration rate = (T0 hr scratch width − T24 hr scratch width)/T0 hr scratch width) × 100%. (**I**) Representative images and (**J**) quantification of the invasive assay, suggesting CD109 loss significantly promoted cancer cell invasion in matrigel invasion assay. 50,000 cells of parental or CD109 KOA431 cells were seeded on a BioCoat™ Matrigel^®^ Invasion Chamber for 24 hours. Cells that invaded through the matrigel-coated membrane were stained with 1% crystal violet, photographed, and counted. All the results are expressed as the mean ± S.D. of three independent experiments. Significance is calculated using a One-Way ANOVA *P < 0.05. **P < 0.01 and ***P < 0.001. Scale bars: 10 μM, 100 μM and 300 μM, as indicated.

Strikingly, CD109-KO cells lose their epithelial tight cell-cell contacts and acquired a mesenchymal phenotype characterized by a spindle-like fibroblastic shape (Figs 4E and 5A). Consistent with these morphological changes, RT-PCR revealed that mRNA levels of fibronectin (FN), vimentin (VIM), a-SMA and collagen were significantly increased in CD109-KO cells (Fig. 6A). In line with these results, as detected by western blots and immunofluorescence microscopy, the mesenchymal proteins such as vimentin, fibronectin, N-cadherin and EMT master transcription factors Zeb2, Snail were markedly increased whereas epithelial protein marker E-cadherin was markedly down-regulated in CD109 KO cells (Fig. 6B,C, Supplementary Fig. S2). To exclude the possibility of clonal artifacts during selection of stable cell lines, we also examined the morphology and the expression of EMT markers in another CD109 KO clone and confirmed similar morphology and EMT marker expression pattern in this KO clone (Fig. 6B,C and Supplementary Fig. S2). These observations are further supported by microarray gene profiling, which demonstrates that genes associated with a mesenchymal phenotype (Supplementary Excel File-1), including EMT master transcription factors and extracellular matrix (ECM) proteins, are the mostly upregulated genes. In contrast, *CDH1*, *ID* and *CLDN12* transcripts are markedly downregulated in CD109 KO cells (Supplementary Excel File-1).

TGF-β is a potential EMT inducer and our group has identified that CD109 is a co-receptor of TGF-β and counteracts TGF-β function^7,8^. We therefore hypothesized that the enhanced EMT response in CD109-KO cells might be due to enhanced TGF-β signaling because of the absence of CD109. Indeed, we observed that the levels of P-Smad2, a downstream effector of TGF-β signaling, were markedly elevated in CD109 KO cells as shown by Western blot (Fig. 6D) and immunofluorescence (Fig. 6E,F), indicating an enhanced TGF-β signaling activity in CD109-KO cells. Collectively, these data suggest that CD109 is required to maintain an epithelial phenotype and supresses the EMT process by inhibiting the TGF-β pathway.

Since enhanced TGF-β signalling and EMT phenotype are usually accompanied by enhanced migration and invasive capacity, we then evaluated the effect CD109 loss on these endpoints by a wound healing assay (Fig. 6G,H) and matrigel invasion assay (Fig. 6I,J). The loss of CD109 increased cellular migration in the wound healing assay by 50% (Fig. 6G,H) and also had a substantial effect on cellular invasiveness, leading to a 3-fold increase of invasion in matrigel trans-well assays (Fig. 6I,J), suggesting an important role of CD109 in the regulation of the invasive behavior of SCC cells. Together, our results show that CD109-KO SCC cells exhibit dramatically increased migratory and invasive behavior.

### CD109-KO cells regain their epithelial phenotype and lose their mesenchymal traits upon the treatment of recombinant human CD109 protein

**Figure 7:**
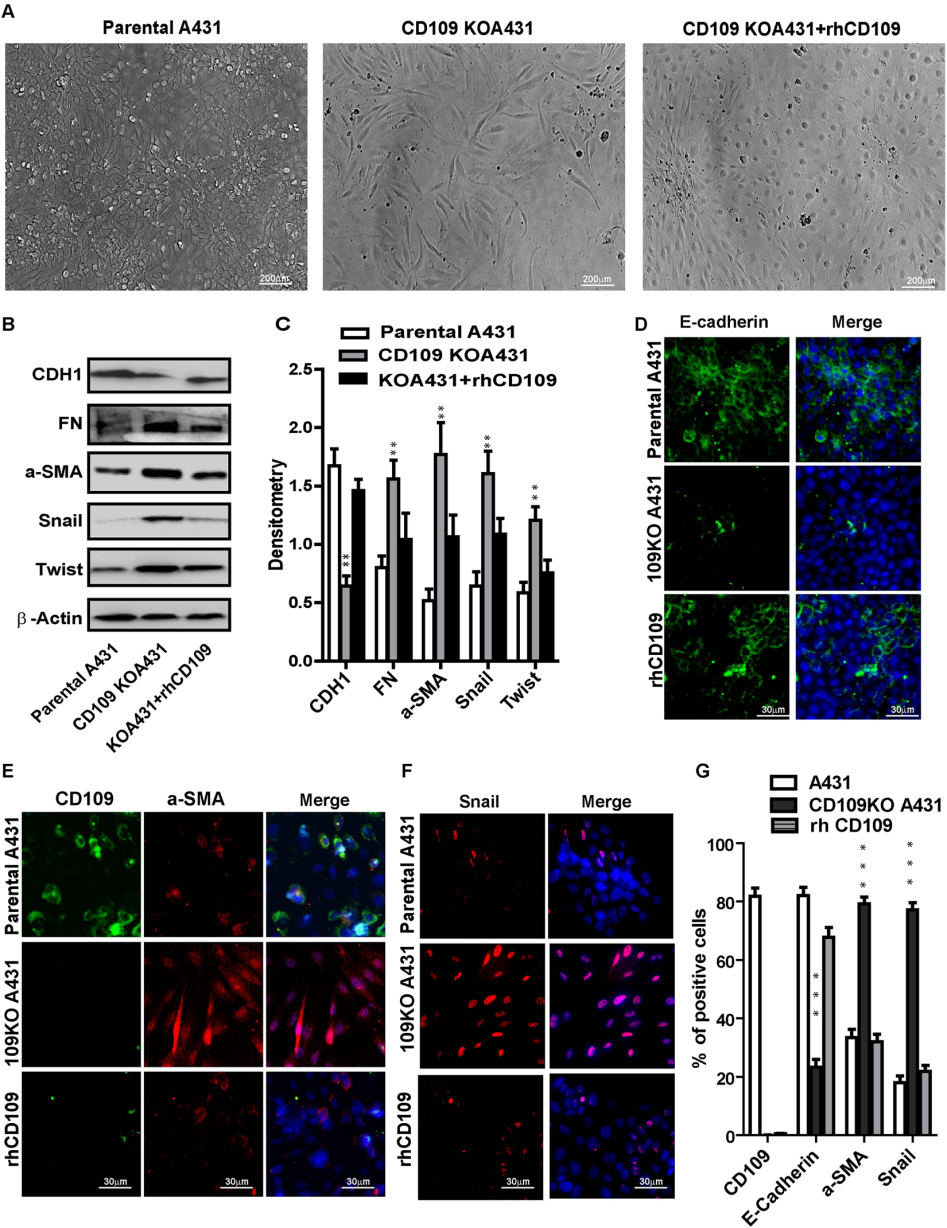
rhCD109 rescue the EMT phenotype of CD109-KO SCC cells. (**A**) Phase contrast microscopy for the morphology of parental A431 cells (Left), CD109-KO A431 cells (Middle), and CD109-KO cells treated with rhCD109 protein (Right), demonstrated that rhCD109 rescued the epithelial phenotype of CD109-KO cells. (**B**) Western blot detection and (**C**) quantification of E-Cadherin (CHD1), fibronectin (FN), alpha-smooth muscle actin (a-SMA), Snail and Twist. The treatment of rhCD109 protein rescue E-cadherin expression and significantly suppressed the expressions of mesenchymal proteins and EMT markers. (**D**–**F**) Representative Immunofluorescence microscopy images and (**G**) quantification for CDH1 (green, **D**), a-SMA (red, **E**), Snail (red, **F**) and DAPI (blue) in parental A431 cells, CD109 KO cells, and CD109-KO cells treated with rhCD109 as indicated, confirming that the treatment of CD109 suppressed EMT markers and rescued the epithelial traits in CD109-KO cells. All the results are expressed as the mean ± S.D. of three independent experiments. Significance is calculated using a One-Way ANOVA *P < 0.05. **P < 0.01 and ***P < 0.001. Scale bars: 20,30 and 200 μm in low and high magnification, as indicated.

To ascertain whether the above observed effects truly depends on CD109 loss and to examine if these effects could be rescued by recombinant human CD109 protein (rhCD109), we treated CD109-KO cells with 10 uM rhCD109 protein. We observed that the KO-cells regained the epithelial phenotype and forfeited their spindle-shaped mesenchymal morphology (Fig. 7A). This was further confirmed by the upregulation of E-cadherin (CDH1) and downregulation of mesenchymal protein FN, a-SMA, and the EMT markers Snail and Twist in the rhCD109 treated SCC cells (Fig. 7B,C). Consistent with this result, immunofluorescence microscopy revealed markedly increased E-cadherin (CDH1) expression (Fig. 7D,G), and decreased expression of a-SMA (Fig. 7E,G) and Snail (Fig. 7F,G), in the rhCD109 treated KO cells. More importantly, the levels of E-cadherin, a-SMA and Snail in rhCD109- treated (rescued) CD109 KO cells reached similar levels as in parental A431 cells, as evidenced by Western blot (Fig. 6B,C) and immunofluorescence microscopy (Fig. 7D–G). The observation that rhCD109 treatment can rescue the epithelial phenotype of CD109 KO-cells demonstrates that the loss of CD109 plays a causative role in mediating the above observed effects and that the phenotype of CD109-KO cells is unlikely to result from potential off-target mutations produced by the CRISPR/Cas9. Overall, our data support the notion that CD109 functions a gatekeeper of epithelial traits of the SCC cells by suppressing the EMT process via inhibition of the TGF-β pathway.

### Loss of CD109 deregulates gene expression profiles and multiple important signaling pathways in SCC cells

**Figure 8:**
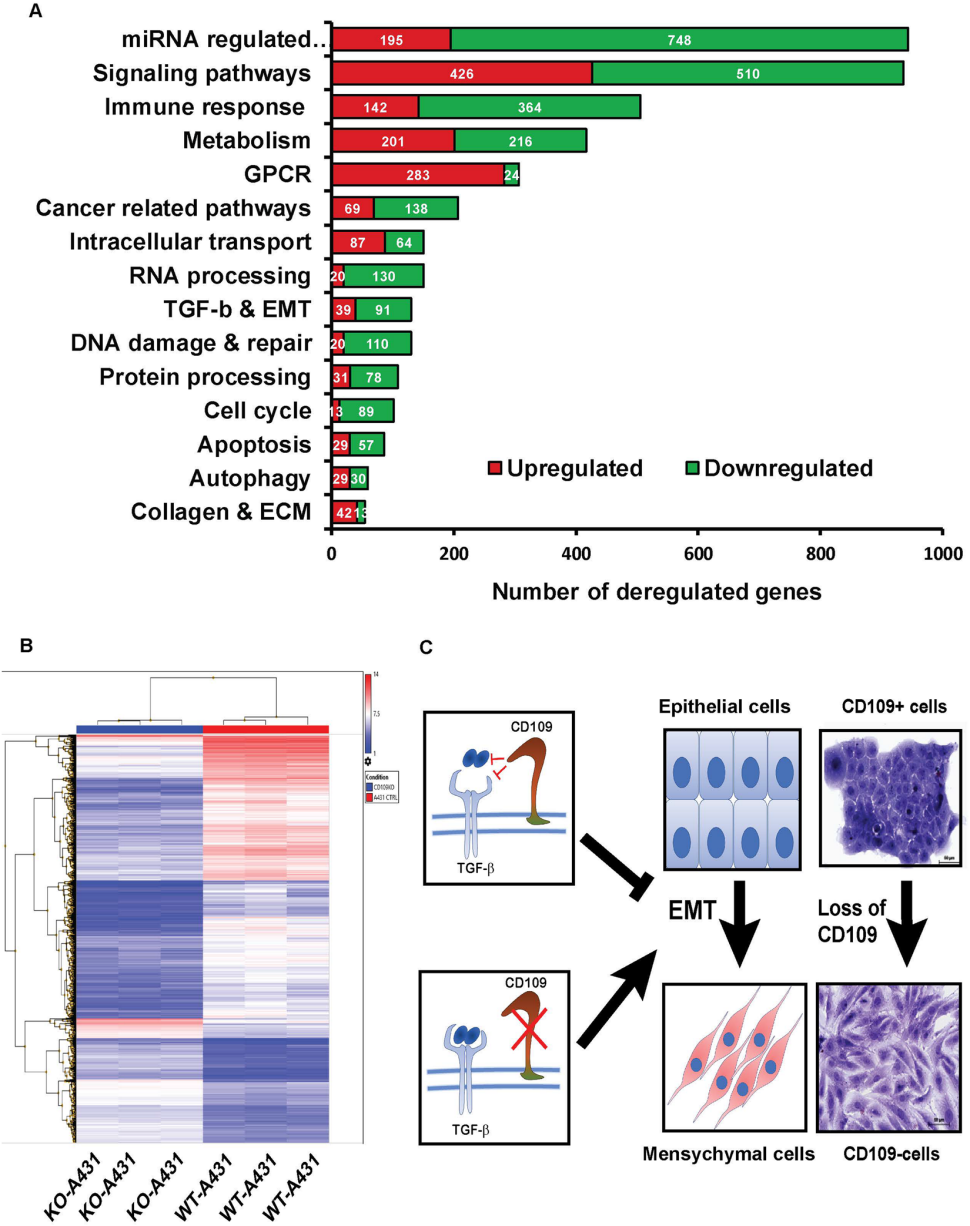
CD109 loss globally affects genes expression and signalling pathways. (**A**) Heat map of differentially regulated genes between CD109-KO and parental A431 cells reveals markedly altered gene expression. Red: higher expression; Blue: lower expression. (**B**) Number of genes deregulated in the fifteen most deregulated pathways identified by a KEGG pathway cluster analysis. (**C**) Proposed mechanism by which CD109 functions as a gatekeeper of the epithelial phenotype: CD109 maintains the epithelial phenotype by inhibiting TGF-β induced EMT. In the absence of CD109, TGF-β-induced EMT is unopposed and results in epithelial cells acquiring a mesenchymal phenotype.

To identify alterations of gene expression associated with the loss of CD109, we performed a mRNA microarray to compare gene expression between CD109-KO and parental A431 cells. A differential expression analysis revealed that CD109-KO cells show pronounced gene expression changes in both directions (down-and up-regulation). The gene list obtained from a class comparison between CD109 KO *versus* parental A431 was filtered based on the criteria of a fold change ≥2 and a false discovery rate (FDR) <0.01. We found 994 up-regulated and 1951 downregulated genes in the CD109-KO cells (biological n = 3, technical triplicate) (Fig. 8B and Supplementary Excel File-1). To identify which genes might contribute to SCC progression, we analyzed the top 20 up- or down-regulated genes upon the loss of CD109 (Supplementary Excel File-1). Interestingly, genes involved in the mesenchymal phenotype (VIM, FN, SPARC), in EMT and the ECM coding proteins, FGF10, PRRX, LOXL1 and FGF pathway were among the most upregulated genes in CD109-KO cells (Supplementary Excel File-1). In contrast, genes associated with epithelial phenotype such as T-cell differentiation protein (Mal), adhesion G protein-coupled receptor F1 (ADGRF1), keratin 15 (KRT15), epithelial splicing regulatory protein (ESRP1), epithelial cell adhesion molecule (EPCAM), epithelial membrane protein 2 (EMP2), and the epithelial-specific marker E-Cadherin (CDH1; an important component for maintaining cell-cell contacts in epithelial cells), were significantly downregulated in CD109-KO cells (Supplementary Excel File-1). To obtain further insight into how the loss of CD109 affects cellular signalling pathways in SCC cells, we subjected the dysregulated genes to a KEGG pathway cluster analysis, which revealed that 15 important pathways are significantly deregulated as a consequence of CD109 loss (Fig. 8A), including signalling pathways associated with: miRNA expression (Fig. 8A); cancer and apoptosis (Fig. 8A and Supplementary Excel File-1); cellular adhesion, collagen and ECM deposition (Fig. 6b and); TGF-β and EMT (Fig. 8A and Supplementary Excel File-1); and matrix metalloproteinases (MMPs) (Supplementary Excel File-1). Together, the above results suggest that loss of CD109 led to dysregulation of genes involved in TGF-β signaling, EMT process, ECM, cell migration/adhesion, apoptosis, remodeling and composition of the cytoskeleton.

## Discussion

CD109 is a GPI-anchored cell surface glycoprotein that is expressed in a wide variety of cell types including skin cells, mesenchymal stem cells and tumor cells^13–17,21,22^. Our group identified CD109 as a component of the TGF-β receptor system and has shown that it negatively regulates TGF-β signaling^7,8^. We have also reported that membrane-anchored CD109 promotes localization of the TGF-β receptors into the caveolar compartment and enhances TGF-β receptor degradation^7,9^. We also demonstrated that CD109 inhibits TGF-β-induced EMT through differential regulation of Smad2/3 and MAP kinase pathways in keratinocyte and SCC cell lines (Bizet *et al*., in preparation). In addition, CD109 can be released from the cell surface by enzymes such as phosphatidylinositol phospholipase C^23^. Furthermore, CD109 can be processed by furinase and cleaved into a soluble CD109 that can be secreted into culture medium^19^. Interestingly, the soluble form of CD109 can also bind TGF-β1 with high affinity and sequester it away from the TGF-β signaling receptors^23^.

Although TGF-β pathway has been implicated in EMT and metastasis, and CD109 counteracts TGF-β function, so far few studies have reported on the relationship among CD109, TGF-β, EMT, migration and invasiveness in SCC cells^24,25^. Here, we initially demonstrate that SCC cells express heterogenous levels of CD109 and that CD109 expression is inversely correlated with TGF-β signaling, EMT process, motility and invasion of SCC cells. We also show that CD109 KO SCC cells acquire a mesenchymal phenotype, and loss of CD109 promotes EMT process, cell motility and invasiveness likely by enhancing the TGF-β/Smad-Sail/Zeb2 axis. Moreover, the effect of the deletion of CD109 in SCC cells can be rescued by rhCD109 as evidenced by the finding that the rhCD109 protein treatment leads to CD109 KO-cells regaining the epithelial phenotype by upregulating E-cadherin and downregulating mesenchymal and EMT marker expressions. These *in vitro* findings are in line with our clinical data from 52 OSCC patient samples which suggest that the expression of CD109 inversely correlates with TGF-β signalling and tumor grades.

It is known that the expression of TGF-β is increased in various tumor types and in general TGF-β expression correlates with poor patient prognosis^26–28^. It is also well documented that TGF-β signaling promotes induction of EMT, invasiveness, and metastasis^29–31^. TGF-β has been shown to have a dual role in cancer progression: it is a tumor suppressor in early cancer development and it switches to become a tumor promoter in late stages of tumor by promoting EMT and metastasis^32,33^. As cancer progresses to advanced stages, cancer cells lose their response to TGF-β–induced growth suppression and become sensitive to the tumor-promoting effects of TGF-β^31^. We hypothesized that when the tumor-promoter function of TGF-β predominates, CD109 can repress TGF-β induced EMT, leading to a decrease in motility and invasion. Indeed, we found that the loss of CD109 enhances TGF-β-induced EMT, up-regulates mesenchymal proteins while downregulating E-cadherin and enhancing migration and invasion in A431 cells. Together, these findings suggest that CD109 plays an important role in the modulation of migration and invasion of SCC cells by blocking TGF-β-mediated EMT. Consistent with this notion, CD109-positive basal-like breast carcinoma shows reduced fat invasion as compared to the CD109-negative counterparts, suggesting that CD109 expression may affect biological properties of cancer cells^15^. Moreover, reduced expression of CD109 on hepatocellular carcinoma tumor vessels is associated with large tumor size, microvascular invasion, and advanced tumor stage and CD109 is an independent risk factor for disease-free survival (*P* = 0.001)^34^.

Loss of E-cadherin not only causes deregulation of cytoskeleton and loss of cell polarity but also leads to increased cell motility and invasiveness^35^. E-cadherin mediates cell-cell adhesion, promotes cell clusters and restricts cell motility toward the extracellular matrix^36^. In the present study, we found that loss of CD109 diminished the E-Cadherin expression while rhCD109 protein rescued the E-cadherin expression in CD109-KO cells, suggesting that CD109 can maintain the E-cadherin expression.

Smads play a crucial role in mediating the intracellular signalling of TGF-β and the TGF-β-Smads2/3 pathway is often aberrantly regulated in cancer^37^. Interestingly, we show that the deletion of CD109 results in an enhanced phosphorylation of Smad2, in an induction of slug, snail and Zeb2 as well as the suppression of E-cadherin with concomitant increase in mesenchymal proteins, such as fibronectin, vimentin and a-SMA. Conversely, the effect of the deletion of CD109 in SCC cells can be rescued by rhCD109, resulting in CD109-KO cells regaining the epithelial phenotype and losing the mesenchymal morphology. This is associated with upregulation of E-cadherin and downregulation of mesenchymal protein fibronectin, vimentin, a-SMA, and the EMT markers.

Our findings highlight a new role of CD109 as a gatekeeper of epithelial cancer cell phenotype and indicate that the underlying mechanism involves inhibition of TGF-β signalling leading to the attenuation of migration and invasion of SCC cells. A schematic model depicting the possible mechanism underlying CD109 function as a gatekeeper of the epithelial phenotype by inhibiting the EMT process is shown in Fig. 8C. In the absence of CD109, TGF-β-induced EMT is unopposed resulting in the loss of epithelial traits and in the acquisition of a mesenchymal phenotype of SCC cells (Fig. 8C). Together, the findings from current study suggest that CD109 is a master regulator of epithelial traits and gatekeeper of epithelial phenotype. CD109 has the potential to play an essential role in SCC progression and is a potential target for therapeutic intervention in SCC *in vitro*.

Although previous work from our laboratory has provided the first evidence to indicate that CD109 is a TGF-β co-receptor and later emphasized its role as a negative regulator of TGF-β signaling^7–9^, there is now increasing evidence to suggest that CD109 is more than a TGF-β co-receptor that inhibits TGF-β signaling. A recent report provides evidence to indicate CD109 enhances EGF signaling while attenuating TGF-β signaling, in SK-MG1 glioblastoma cells^38^. Furthermore, CD109 has been proposed to act as a novel pro-metastatic factor that can regulate metastatic ability in lung adenocarcinoma by modulating Jak-Stat3 activity *in vivo*^39^. It is therefore possible that the consequences of loosing CD109 in cancer progression and metastasis are more complex. While CD109 has the ability to inhibit TGF-β-induced EMT and to enhance migration of cancer cells, it may promote tumor initiation and metastasis by enhancing the EGF or the JAK-STAT3 pathway. A precise definition of the molecular pathways regulated by CD109 and the consequences of their dysregulation in cancer progression and metastasis remain to be determined.

## Methods

### Study population

SCC samples were surgically removed at the Department of Otorhinolaryngology Head and Neck-Surgery (McGill University). This study was approved by the Research Ethics Committee at the Jewish General Hospital (Protocol #11-093) and carried out in accordance with the approved guidelines and regulations. All experimental protocols were approved by Research Review Office (RRO, Canada). Patients were advised of the procedures and provided written informed consent. The eligibility criteria included previously untreated patients, without a second primary tumor and submitted for treatment in the same institution.

### Tissue microarray (TMA) platform

Tissue cores with a dimension of 1.0 mm from each specimen were punched and arrayed in duplicate on a recipient paraffin block using a Tissue Microarrayer (Beecher Instruments®, Silver Springs, MD, USA). After cutting sections from the recipient block and transferring these with adhesive tape to coated slides for subsequent UV cross-linkage (Instrumedics Inc®, Hackensack, NJ, USA), the slides were dipped in a layer of paraffin to prevent oxidation and stored in a freezer at −20 °C.

### Immunohistochemistry (IHC) analysis and scoring

IHC was carried out on TMA as described earlier^40^. Incubations with the primary antibodies were conducted overnight at 4 °C for: mouse monoclonal anti-CD109 (Cat. No. 556039, BD Biosciences). TGFBR1/ALK5 Antibody (aa281-295, LS-C312882), anti-P-Smad2 (Cat. No. ab188334, Abcam) and anti-TGFβ1 (Cat. No. ab92486, Abcam). The sections were washed and incubated with secondary antibodies (Advanced ™ HRP Link, DakoCytomation, K0690, Denmark) followed by the polymer detection system (Advanced ™ HRP Link, DakoCytomation). Reactions were developed with a solution containing 0.6 mg/mL of 3,3′-diaminobenzidine tetrahydrochloride (DAB, Sigma, St Louis, MO, USA) and 0.01% H_2_O_2_ and then counter-stained with Mayer’s hematoxylin. Positive controls (a tissue known to contain the antigen under study) were included in all reactions in accordance with manufacturer’s protocols. The negative control consisted in omitting the primary antibody and incubating slides with PBS and replacing the primary antibody with normal serum. The IHC reactions were performed in duplicate on different TMA levels, representing four-fold redundancy for each case. The second slides were 25 sections deeper than the first, resulting in at least 250 μm of distance between the two sections with different cell samples for each tumor.

IHC analysis was performed blindly to the clinical aspects and conducted by two independent certified pathologists. Each core was scanned in low power field to choose the most stained area predominant in at least 10% of tumor cells. The presence of a clearly visible dark brown precipitation was considered positive for immunostaining. Slides were analyzed as previously described^41^ in accordance to the staining intensity: 0, no visible reaction; 1, weak expression, 2: strong positivity. For statistical analysis, the samples were categorized into groups: negative and positive cases.

### Cell culture and reagents

Human squamous carcinoma cell A431 (Cat. No. ATCC® CRL1555™) and FaDu (ATCC® HTB-43™) cell lines were purchased from the American Type Culture Collection. A431 cells were maintained in Dulbecco’s modified Eagle’s medium (DMEM; Gibco Life Technologies, USA Cat No. 11995-065) supplemented with 10% fetal bovine serum (FBS; Gibco Life Technologies, Canada; Cat. No.12483-020) at 37 °C in 5% CO_2_. FaDu cells were cultured in MEM media supplemented with 10% fetal bovine serum (FBS) and incubated at 37 C in humidified atmosphere of air containing 5% CO_2_.

The recombinant human CD109 protein used in this study was obtained from R& D systems, Catalog Number: 4385-CD, which is the soluble CD109 protein of 1268 amino acids with 141KDa molecular weight.

### Fluorescence-activated cell sorting

80% confluent SCC cells were detached, harvested and resuspended in cold PBS, then were stained with antibodies CD109-PE (clone: W7C5, Cat. NO. 323305, Biolegend, USA). Then, SCC cells were sorted to subtype cells according to CD109 expression level by BD FACSAria III (BD Biosciences) at a flow rate of less than 3000 cells/s. Gate sets as 10% CD109 highest as CD109^high^, 10% lowest as CD109^Low^, and CD109^med^ is defined between 10% CD109 high and 10%CD109 low.

### Immunofluorescence staining

Cells were fixed in 4% paraformaldehyde (w/v) for 15 min, and permeabilized in PBS/0.3% Triton X-100 for another 15 min. Cells were washed with PBS and blocked in 2% BSA for 1 h. Primary antibodies against anti-CD109 (C-9) (Cat. No. sc-271085, Santa Cruz Biotechnology); anti-Snail (Cat. NO. ab5351, abcam), anti-a-SMA (Cat. NO. ab5694, abcam), anti-P-Smad2 (Cat. NO. mAb #3108, cell signalling), anti-Ki67 (Cat. No. sc-23900, Santa Cruz) were added to cells at 1:300 dilution in 2% BSA and incubated overnight at 4 °C. Cells were washed with PBS and labeled for 1 h with fluorophore-conjugated secondary antibodies in 1:500 dilution;(Alexa Fluor 594-goat anti-rabbit (A11037, life technology), Alexa Fluor 488 goat-anti-mouse, A11029, life technology). Cells were washed with PBS and the slides were mounted on coverslips with Fluoroshield mounting medium with DAPI (Cat. No. ab104139, Abcam). Cells were visualized using an LSM780 confocal microscope or Olympus microscope IX71.

### Western blot analysis

The proteins were extracted from the whole cell lysates using RIPA cell lysis buffer and the protein concentration was determined by the Bradford reagent. In total, 20 μg of the extracted total cellular protein from each sample were separated via SDS-PAGE, and transblotted onto EMD Millipore Immobilon™-P PVDF Transfer Membranes (EMD Millipore Cat No.: IPVH00010, USA). Western blot analyses were conducted with the following antibodies: mouse monoclonal anti-CD109 (Cat. No. 556039, BD Biosciences), anti-fibronectin (Cat. No. 610078, BD Biosciences), anti-Vimentin (D21H3) XP^®^ Rabbit (Cat. No. 5741. Cell signaling); Anti-ZEB2 (Cat. No. sc-271984, Santa Cruz Biotechnology); rabbit polyclonal anti-Snail (Cat. No. ab5351, Abcam), anti-twist (Cat. No. ab505181; Abcam), anti-TGFBR1 (ALK5) (Cat. No. AHO1552, Invitrogen), anti-N Cadherin (Cat. No. ab18203, Abcam), anti-E-Cadherin (Cat. No. ab216783, Abcam), Anti-P-Smad2 (Cat. No. mAb #3108, cell signalling); Anti-P-Smad2/3 (Cat. No. MAB8935, R&D system, and anti- Slug (Cat. No. 9585S Cell signaling), anti-β-actin antibodies (Cat. No. sc-47778, Santa Cruz Biotechnology).

### Wound healing assay

A431 CD109^high^, CD109^med^ and CD109^low^ cells were seeded at a density of 6 × 10^5^ cells/well into Costar® 6 Well Clear TC-Treated Multiple Well Plates (Product #3516, Corning Inc, USA) and cultured for ~48 h or until the cells had reached ~90% confluency. Cells were then pre-incubated with serum-free medium (SFM) for 24 h to inhibit cell proliferation^42^. The monolayer of A431 cells were scratched across the centre with a sterile 200 μl pipette tip to create a cell-free line. The culture medium was aspirated and washed three times to remove cellular debris. The culture plates were replenished with serum free DMEM in the absence or presence of 100 pm TGF-β1 (Cat. No. 7754-BH-005, R&D). Photographs were taken immediately (0 h) and 24 h after the scratch and Image-J software was used to measure the width of the wound area. The experiments were repeated 3 times. Cell migration was expressed as percentage of the scratch area filled by migrating cells at 24 h post scratch: migration rate = (T0 hr scratch width − T24 hr scratch width)/T0 hr scratch width) × 100%.

### Matrigel invasion assay

The Matrigel invasion assay was done by using the BD Biocoat Matrigel Invasion Chamber (Cat. No. 354480; pore size: 8 mm, 24-well; BD Biosciences) according to the manufacturer’s protocol. Briefly, the control inserts or matrigel-coated inserts were rehydrated with plain DMEM for 2 h before use. Cells (5 × 104 cells) in 500ul Dulbecco’s modified Eagle’s medium without serum were seeded on the upper chamber; the lower chamber was filled with DMEM supplemented with 10% FBS as chemoattractant. After 24 hours, cells on the upper side of the membrane were wiped off; cells on the lower side of the membrane were fixed for 10 min by cold methanol and stained with 0.1% crystal violet solution for 20 min and washed with PBS 3 times. Cell numbers were counted using an inverted microscope at ×200 magnification with 10 fields of view, and the mean values were taken as the invasive cell number. All assays were done in triplicate for at least three independent experiments

### RNA isolation and quantitative real-time PCR

Total RNA was isolated from cultured cells using RNeasy mini Kit (Cat. No. 74104, QIAGEN). Reverse transcription (RT) was done on 1 μg of cellular total RNA using the MML-V reverse transcriptase (Invitrogen). Quantitative real-time PCR (qPCR) was performed using the Platinum SYBR Green SuperMix (Invitrogen) and an ABI Prism 7500 Real-Time PCR apparatus (Applied Biosystems). Primer sets used were as follows:

human GAPDH: forward primer 5′-GACAACTTTGGTATCGTGGAAGG-3′;

reverse primer 5′-AGGGATGATGTTCTGGAGAGCC-3′;

human Vimentin: forward primer: 5′-GGACCAGCTAACCAACGACA-3′

reverse primer: 5′-AAGGTCAAGACGTGCCAGA-3′;

human Fibronectin: forward primer: 5′-ACAACACCGAGGTGACTGAGAC-3′

reverse primer: 5′-GGACACAACGATGCTTCCTGAG-3′

human α-SMA: forward primer: 5′-CCGACCGAATGCAGAAG GA-3′

reverse primer: 5′-ACAGAGTATTTGCGCTCCGAA-3′

human Collagen 1A1 (Col1A1):

forward primer 5′-GATTCCCTGGACCTAAAGGTGC-3′,

reverse primer 5′-AGCCTCTCCATCTTTGCCAGCA-3′

human Collagen3A1 (Col3A1):

forward primer 5′-TGGTCTGCAAGGAATGCCTGGA-3′

reverse primer 5′-TCTTTCCCTGGGACACCATCAG-3′

GAPDH was used as an internal standard for data calibration. The 2-ΔΔCt formula was used for the calculation of differential gene expression.

### Generation of knockout cell line with CRISPR/Cas9

The experimental approach comprised generation of stable CD109 KO cell lines by follow the nature protocol^20^. Guide RNA sequences for CRISPR/Cas9 were designed at CRISPR design web site (http://crispr.mit.edu/), provided by the Feng Zhang Lab^20^. Insert oligonucleotides for human CD109 gRNA #1 and #2 (targets the exon 1 and exon2 of CD109 gene) are 5′-CACCGCCTCCGGGCCTGATG ATCCC-3′/5′-CACCGACTATTGGGGTGGAGCTTC-3′, respectively. The complementary oligo-nucleotides for guide RNAs (gRNAs) were annealed and cloned into pX458 CRISPR/Cas9-GFP vector (Addgene, Cambridge, MA). SCC cells were transfected with either pX458/gRNA #1 or pX458/gRNA #2 using lipofectamine 200, according to the manufacturer’s instructions. Two days after transfection, cells were for GFP positive cells for expansion. After two weeks expansion, these GFP positive cells stained for CD109 and sorted per single CD109 negative cell into per well in a 96-well plate. The single CD109 negative cell was used to generate CD109 KO single cell clone, and the CD109 sequences were analyzed with T7 endonuclease (T7E1) assay, DNA sequencing and Western blot.

### Gene microarrays and data analysis (affymetrix expression console™ software)

Tumor cell monolayers growing in complete medium were used for RNA extraction when 75% confluent. Total RNA was isolated and purified using an RNeasy Mini Kit (Qiagen) according to manufacturer protocol. RNA integrity was confirmed by electrophoresis on an Agilent 2100 instrument, and only samples with RINs of >9 were analyzed. A total of 23,937 probe sets, which interrogate 23,256 transcripts representing 20,201 human genes were included in the Affymetrix GeneChip of Clariom_S_human GeneChip array (Affymetrix Inc., Santa Carla, CA, USA). RNAs were labeled and hybridized to Affymetrix Genechips Clariom_S_human GeneChip array. Labeling, hybridization, and scanning were carried out by Genome Quebec. Pre-processing, normalization, and background correction were carried out using Gene Expression Console software (Affymetrix); then the obtained chp files were analyzed by Transcriptome Analysis Console 4.0 (TAC) to identify genes that were significantly regulated, and a filtered gene list was generated with p value (p < 0.01) and fold change >+2 or <−2 at false discovery rate as less than 0.01. Differentially expressed genes screened by Volcano Plot filtering were further investigated to determine the pathways using the latest Kyoto Encyclopedia of Genes and Genomes (KEGG) database^43^. The p-value denotes the significance of the pathway-term enrichment, and the p-value cut-off was set at 0.05. Microarray results were deposited on the GEO Database (accession number GSE117475).

### Statistical analysis

All values are expressed as mean of at least 3 independent experiments ± SD. Comparisons between two groups were analyzed by a two-tailed Student’s-test, and comparisons between more than two groups were analyzed by one-way ANOVA. Chi-square has been used to find the significance of study parameters on categorical scale between two or more groups. A value of P < 0.05 was considered statistically significant. All analyses were performed with Prism GraphPad.

## Supplementary information

Spupplementary Data
