# Supplementary material for: CD109 acts as a gatekeeper of the epithelial trait by suppressing epithelial to mesenchymal transition in squamous cell carcinoma cells in vitro
Source: Sci Rep. 2019 Nov 6;9:16317. doi: 10.1038/s41598-019-50694-z (PMC6834570; doi:10.1038/s41598-019-50694-z)
Supplement: Supplementary file 1 — Spupplementary Data [file 41598_2019_50694_MOESM1_ESM.pdf]

CD109 acts as a gatekeeper of the epithelial trait by suppressing epithelial to mesenchymal transition in squamous cell carcinoma cells *in vitro*

Shufeng Zhou [1], Sabrina Daniela da Silva[2], Peter M. Siegel[3] and *Anie Philip[1]

[1]Division of Plastic Surgery, Department of Surgery

[2]Department of Otolaryngology – Head and Neck Surgery

[3]Rosalind and Morris Goodman Cancer Research Centre

Faculty of Medicine, McGill University, Montreal

Correspondence to: Anie Philip, **Email: anie.philip@mcgill.ca**

Montreal General Hospital 1650 Cedar Avenue Room C10-148.4

Montreal, Quebec H3G 1A4

Tel: 514-934-1934 Ext 44535

Supplementary materials

Supplemental Table 1:Clinical characteristics of OSCC patients

Variable	Category	Paraffin-embedded samples n(%)
Age	< 50 year	8 (15.4)
	≥ 50 year	44 (84.6)
Gender	Male	31 (59.6)
	Female	21 (40.4)
Clinical stage	T1+T2	32 (61.5)
	T3+T4	20 (38.5)
Lymph nodes	N0	40 (76.9)
	N+	12 (23.1)
Recurrence or metastasis	No	40 (76.9)
	Yes	12 (23.1)
Status	Alive	45 (86.5)
	Dead	7 (13.5)

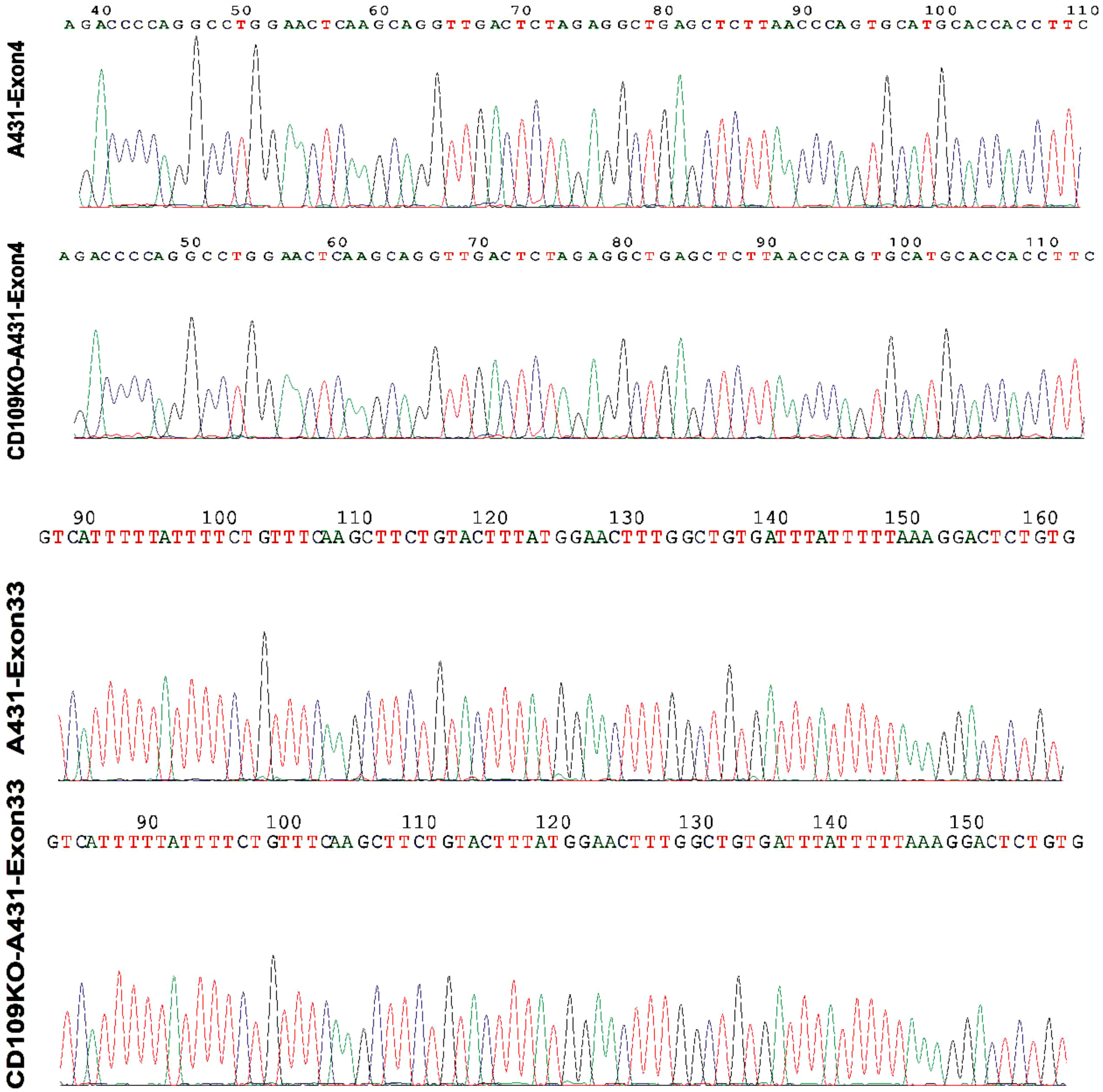

Supplementary Figure S1. Sequencing of PCR amplicons from the exons of CD109

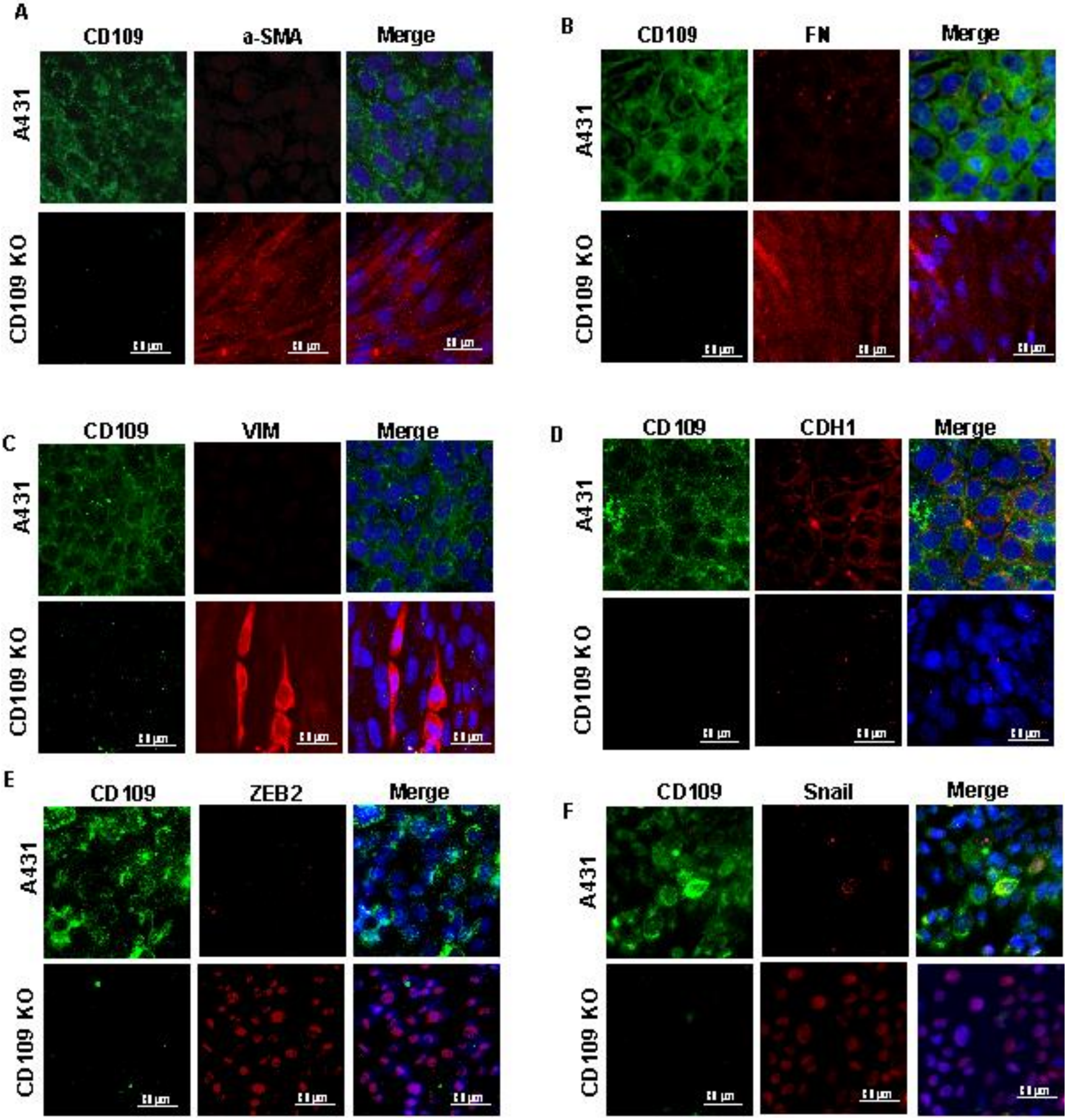

Supplementary Figure S2 Immunofluorescence microscopy of A431 cell and CD109 KO cells stained for CD109 (green), a-SMA (A), Fibronectin (B), VIM (C), CDH1 (D), ZEB2 (E), Snail (F) and DAPI (blue) confirmed that the loss of CD109 markedly enhanced EMT markers and suppressed epithelial traits.

Figure 2C western blots

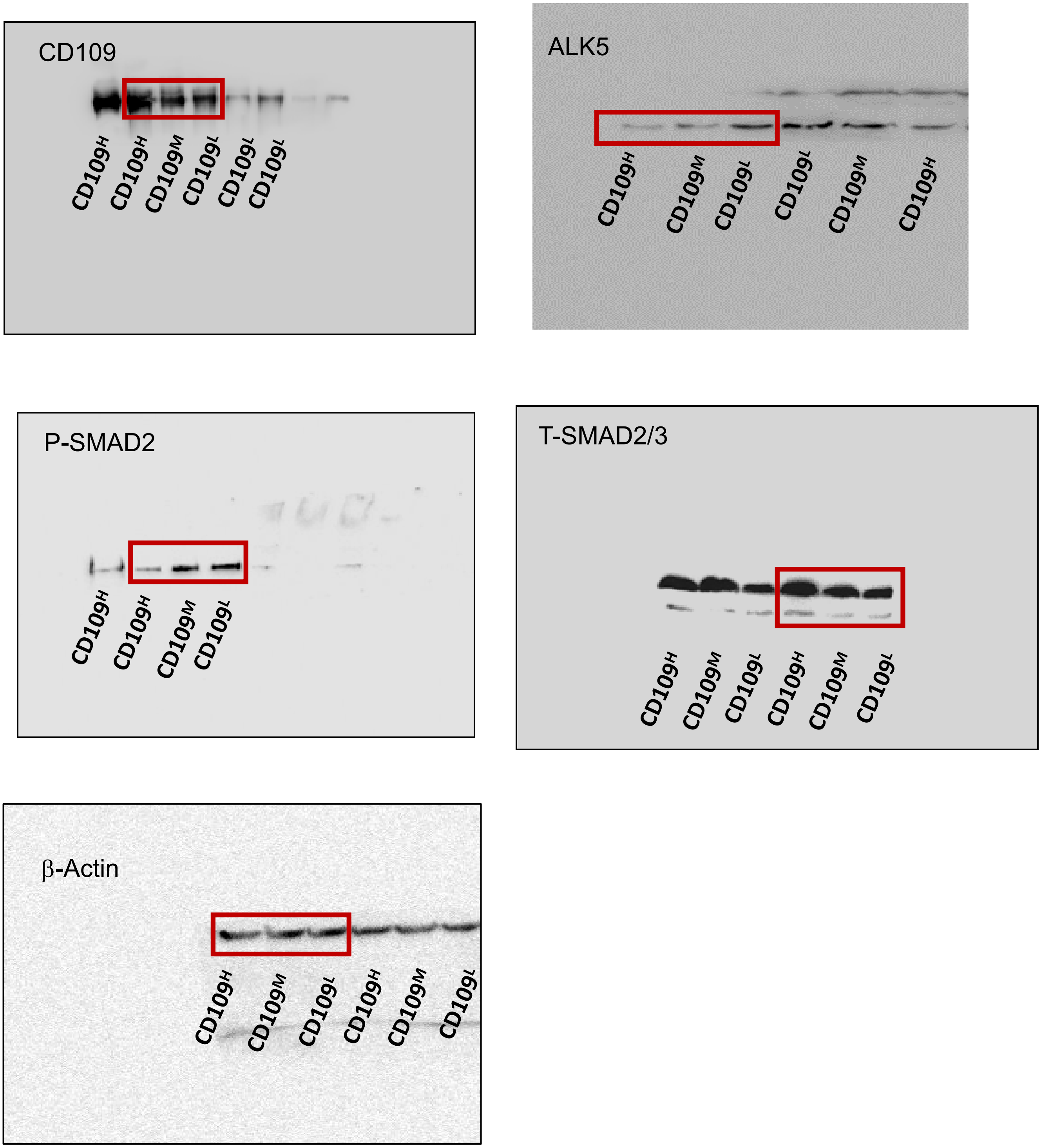

Figure 2 E

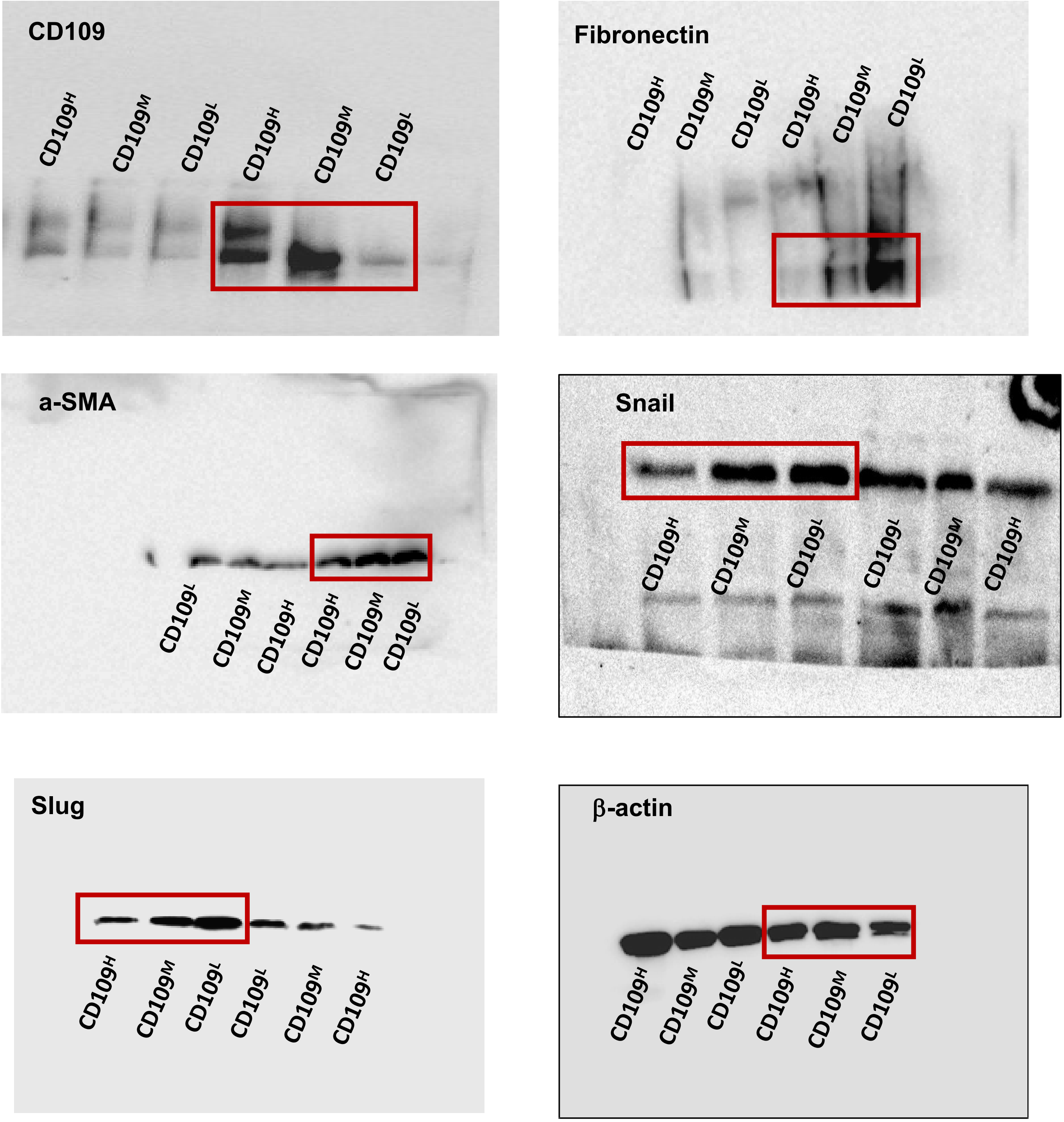
CD109
CD109^{H} CD109^{M} CD109^{L} CD109^{H} CD109^{M} CD109^{L}
Fibronectin
CD109^{H} CD109^{M} CD109^{L} CD109^{H} CD109^{M} CD109^{L}
a-SMA
CD109^{L} CD109^{M} CD109^{H} CD109^{H} CD109^{M} CD109^{L}
Snail
CD109^{H} CD109^{M} CD109^{L} CD109^{L} CD109^{M} CD109^{H}
Slug
CD109^{H} CD109^{M} CD109^{L} CD109^{L} CD109^{M} CD109^{H}
β-actin
CD109^{H} CD109^{M} CD109^{L} CD109^{H} CD109^{M} CD109^{L}

Figure. 3B

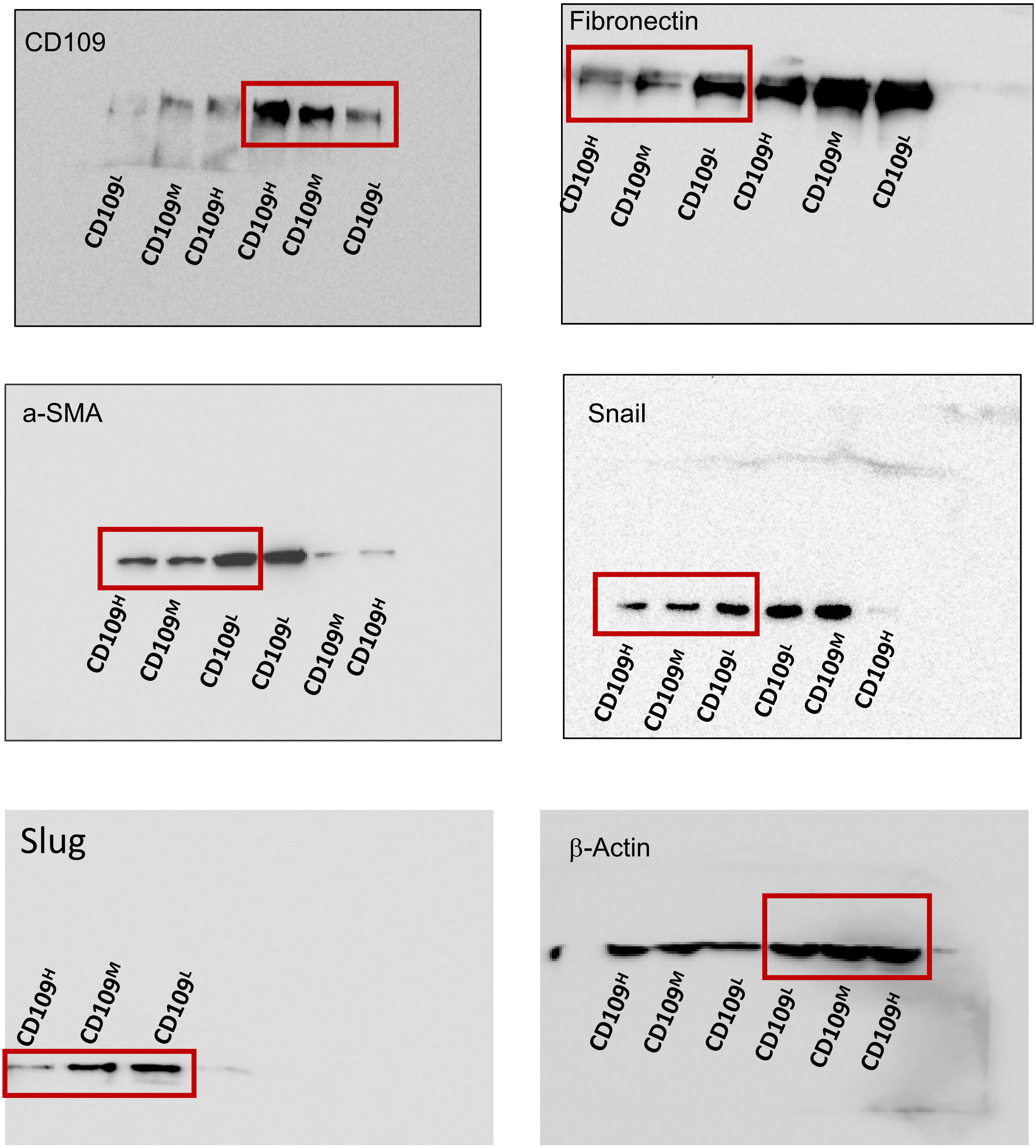
CD109
CD109^{L} CD109^{M} CD109^{H} CD109^{H} CD109^{M} CD109^{L}
Fibronectin
CD109^{H} CD109^{M} CD109^{L} CD109^{H} CD109^{M} CD109^{L}
a-SMA
CD109^{H} CD109^{M} CD109^{L} CD109^{L} CD109^{M} CD109^{H}
Snail
CD109^{H} CD109^{M} CD109^{L} CD109^{L} CD109^{M} CD109^{H}
Slug
CD109^{H} CD109^{M} CD109^{L}
β-Actin
CD109^{H} CD109^{M} CD109^{L} CD109^{L} CD109^{M} CD109^{H}

Figure.4D

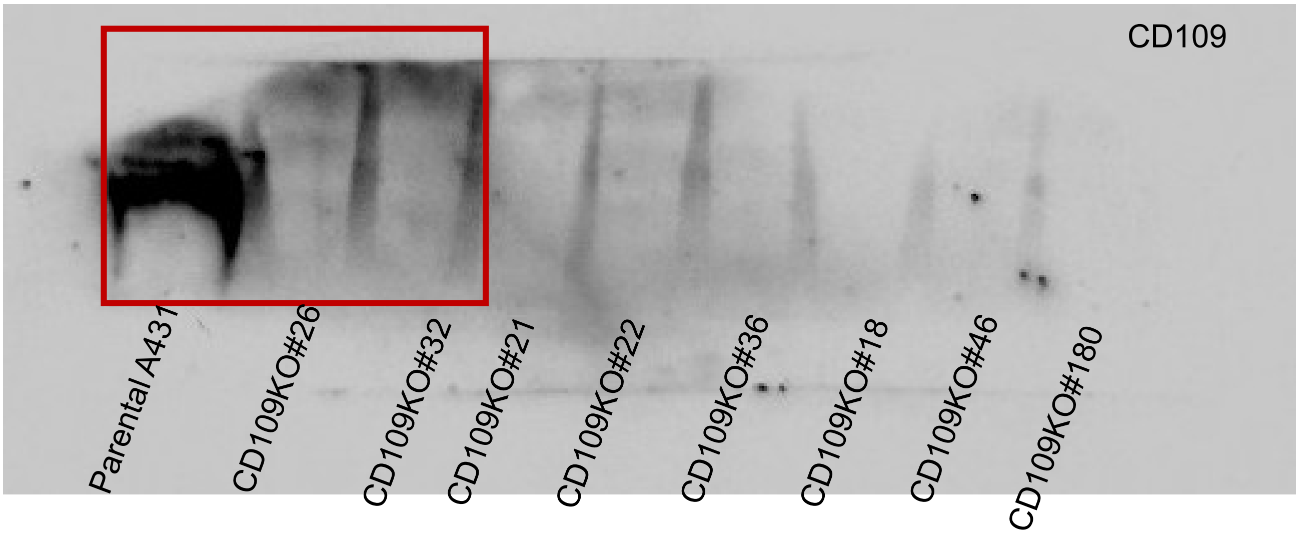
CD109
Parental A431
CD109KO#26
CD109KO#32
CD109KO#21
CD109KO#22
CD109KO#36
CD109KO#18
CD109KO#46
CD109KO#180

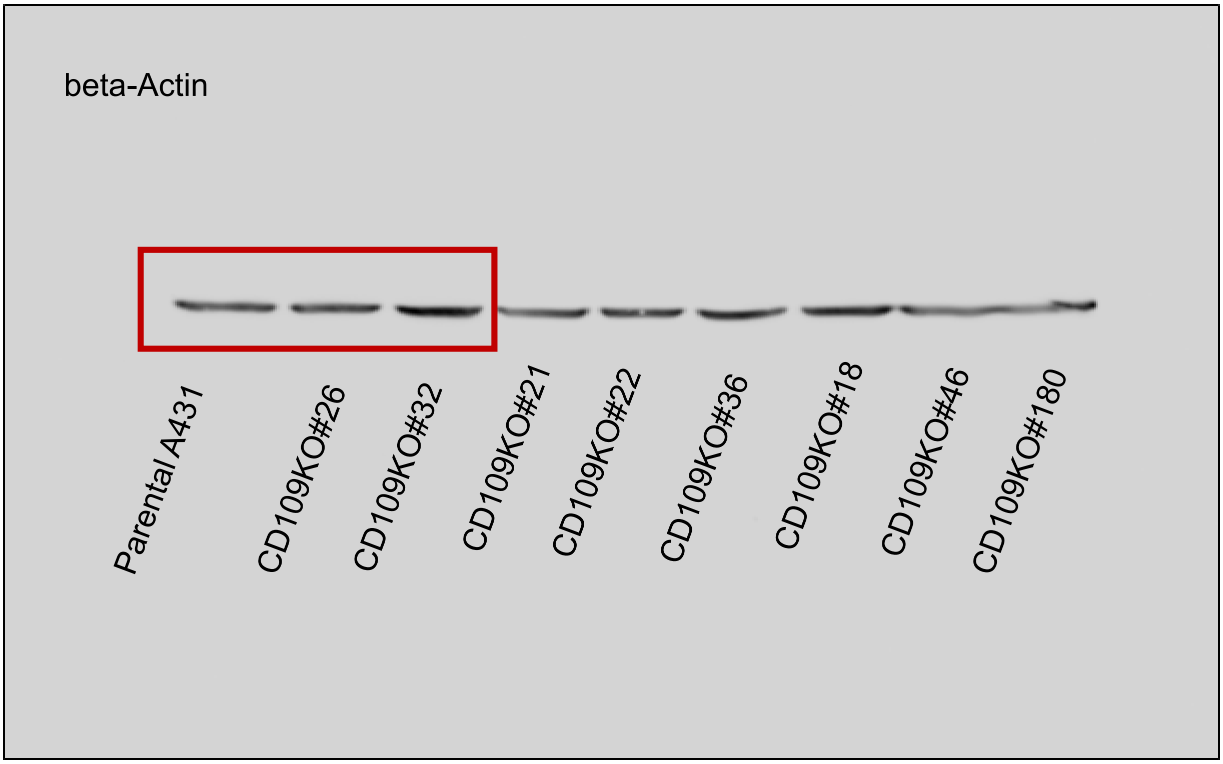
beta-Actin
Parental A431
CD109KO#26
CD109KO#32
CD109KO#21
CD109KO#22
CD109KO#36
CD109KO#18
CD109KO#46
CD109KO#180

Figure.5H

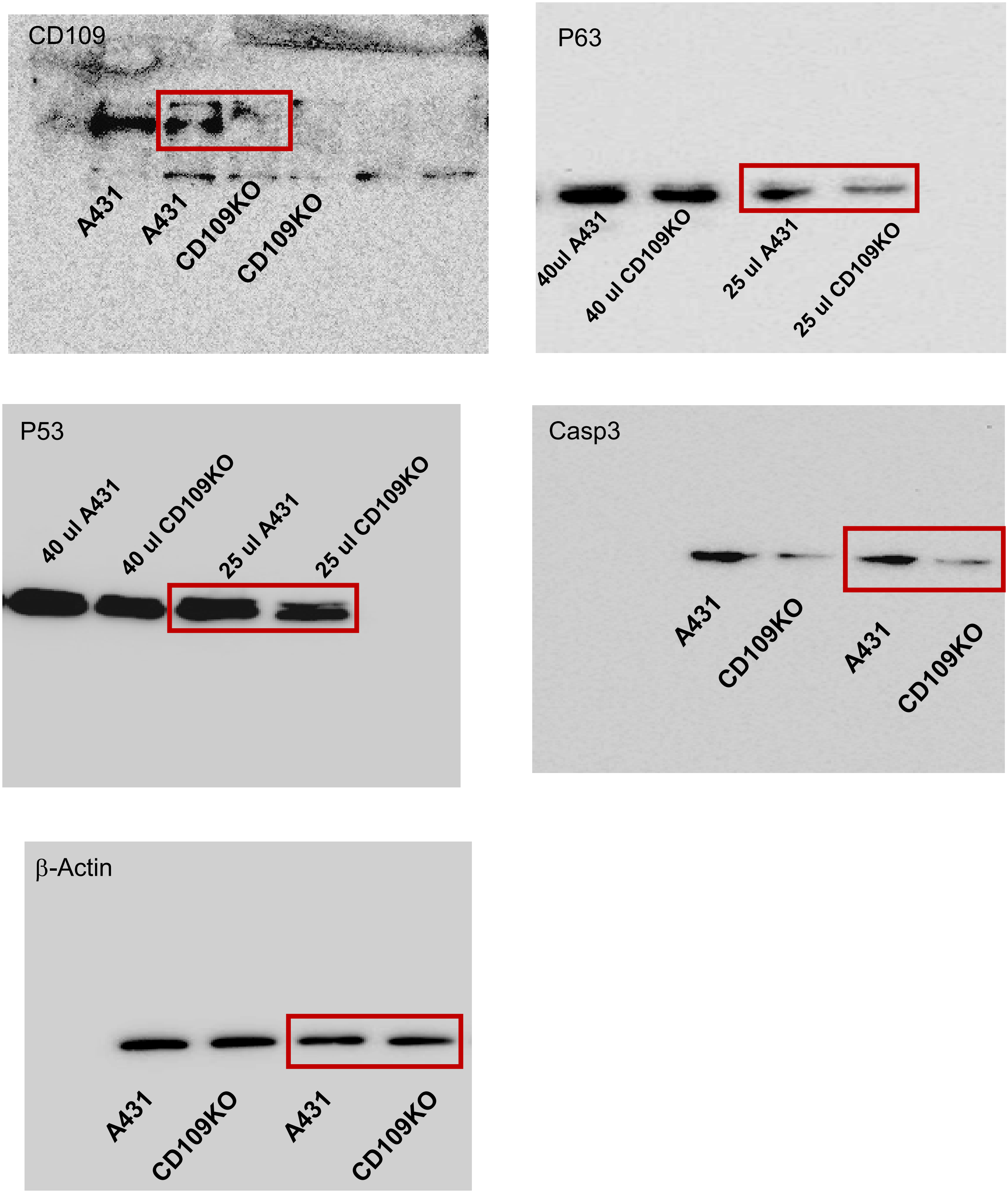

CD109
A431
A431
CD109KO
CD109KO
P63
40ul A431
40 ul CD109KO
25 ul A431
25 ul CD109KO
P53
40 ul A431
40 ul CD109KO
25 ul A431
25 ul CD109KO
Casp3
A431
CD109KO
A431
CD109KO
β-Actin
A431
CD109KO
A431
CD109KO

Figure. 6 B

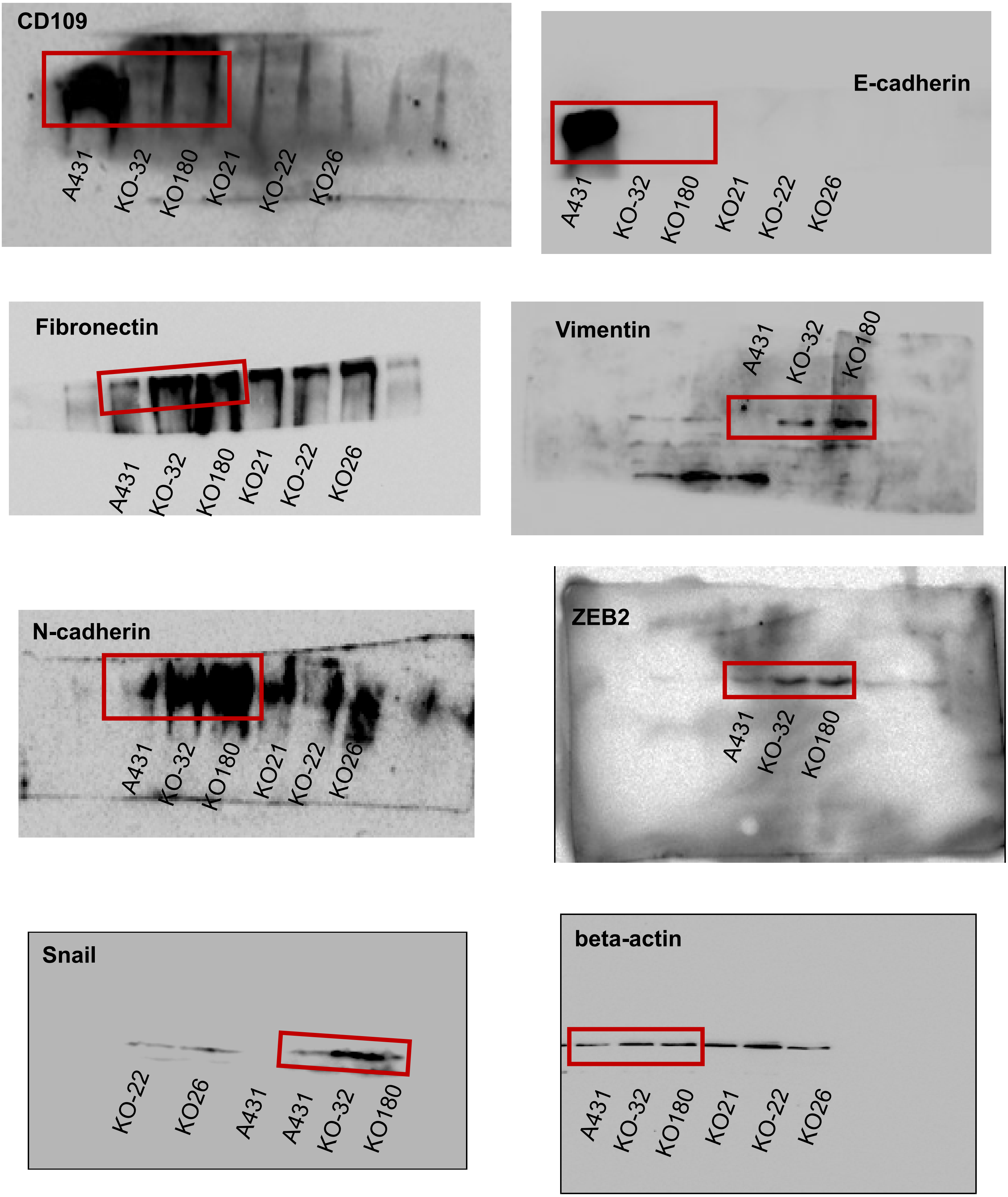
CD109
A431
KO-32
KO180
KO21
KO-22
KO26
E-cadherin
A431
KO-32
KO180
KO21
KO-22
KO26
Fibronectin
A431
KO-32
KO180
KO21
KO-22
KO26
Vimentin
A431
KO-32
KO180
N-cadherin
A431
KO-32
KO180
KO21
KO-22
KO26
ZEB2
A431
KO-32
KO180
Snail
KO-22
KO26
A431
A431
KO-32
KO180
beta-actin
A431
KO-32
KO180
KO21
KO-22
KO26

Figure. 6D

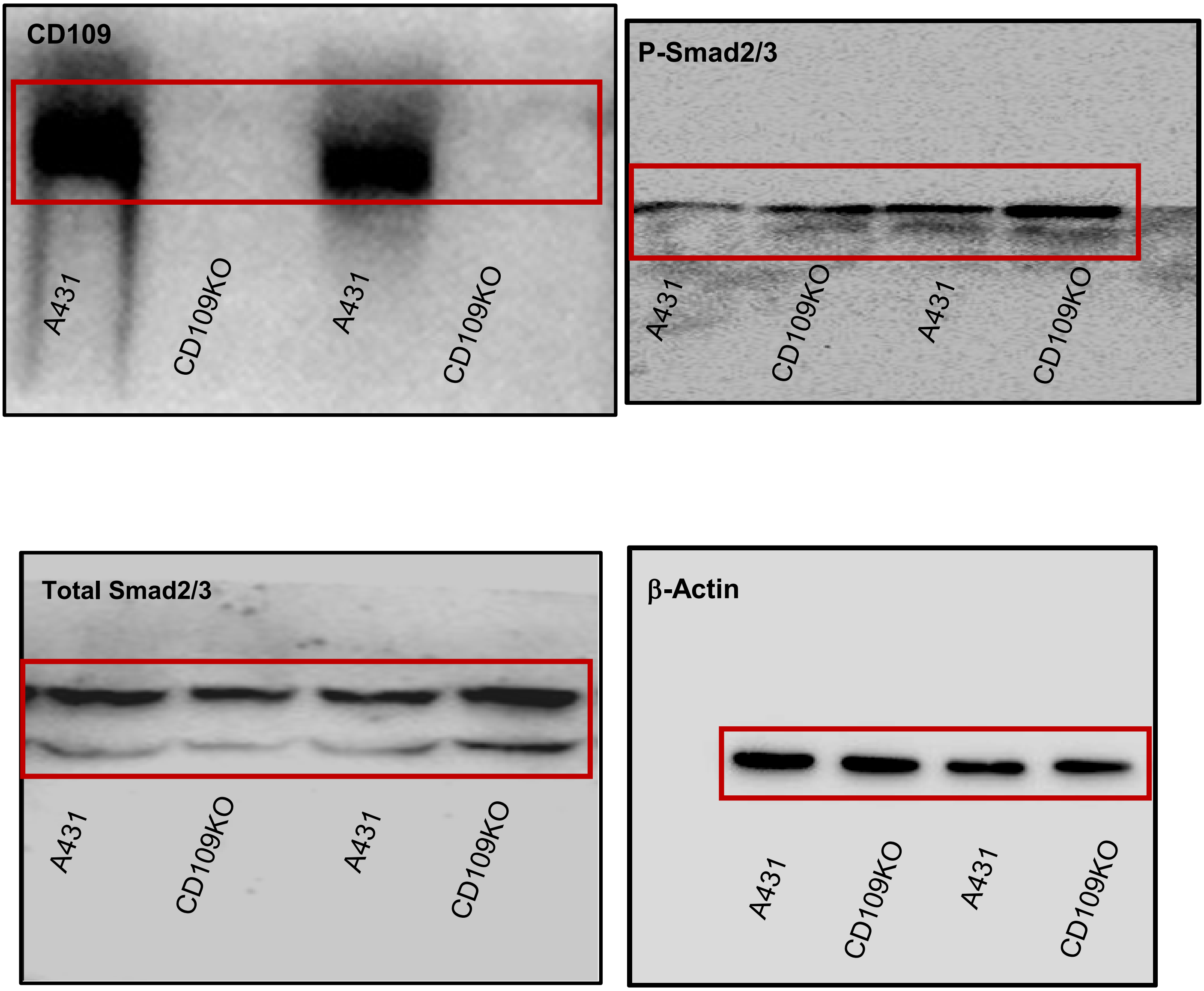
CD109
A431
CD109KO
A431
CD109KO
P-Smad2/3
A431
CD109KO
A431
CD109KO
Total Smad2/3
A431
CD109KO
A431
CD109KO
β-Actin
A431
CD109KO
A431
CD109KO

Figure. 7 B

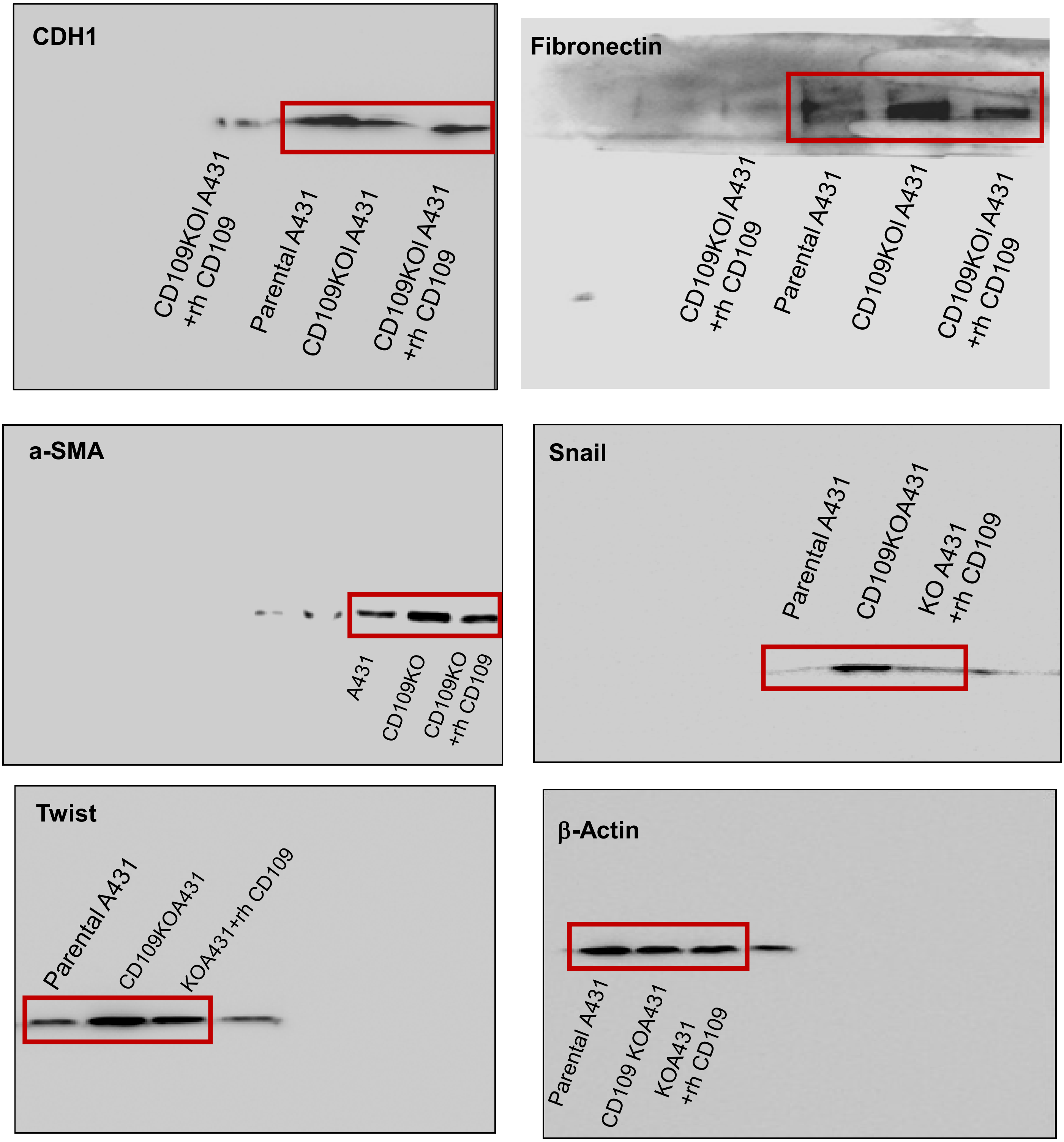

CDH1
CD109KOI A431 +rh CD109
Parental A431
CD109KOI A431
CD109KOI A431 +rh CD109
Fibronectin
CD109KOI A431 +rh CD109
Parental A431
CD109KOI A431
CD109KOI A431 +rh CD109
a-SMA
A431
CD109KO
CD109KO +rh CD109
Snail
Parental A431
CD109KOA431
KO A431 +rh CD109
Twist
Parental A431
CD109KOA431
KOA431+rh CD109
β-Actin
Parental A431
CD109 KOA431
KOA431 +rh CD109
